# Identification and validation of a novel predictive signature based on hepatocyte-specific genes in hepatocellular carcinoma by integrated analysis of single-cell and bulk RNA sequencing

**DOI:** 10.1186/s12920-024-01871-1

**Published:** 2024-04-23

**Authors:** Yujian He, Wei Qi, Xiaoli Xie, Huiqing Jiang

**Affiliations:** Department of Gastroenterology, The Second Hospital of Hebei Medical University, Hebei Key Laboratory of Gastroenterology, Hebei Institute of Gastroenterology, Hebei Clinical Research Center for Digestive Diseases, Shijiazhuang, Hebei China

**Keywords:** Hepatocellular carcinoma, Single-cell RNA sequencing, Hepatocyte-specific genes, Prognosis, Differentiation trajectory

## Abstract

**Background:**

Hepatocellular carcinoma represents a significant global burden in terms of cancer-related mortality, posing a substantial risk to human health. Despite the availability of various treatment modalities, the overall survival rates for patients with hepatocellular carcinoma remain suboptimal. The objective of this study was to explore the potential of novel biomarkers and to establish a novel predictive signature utilizing multiple transcriptome profiles.

**Methods:**

The GSE115469 and CNP0000650 cohorts were utilized for single cell analysis and gene identification. The Cancer Genome Atlas (TCGA) and International Cancer Genome Consortium (ICGC) datasets were utilized in the development and evaluation of a predictive signature. The expressions of hepatocyte-specific genes were further validated using the GSE135631 cohort. Furthermore, immune infiltration results, immunotherapy response prediction, somatic mutation frequency, tumor mutation burden, and anticancer drug sensitivity were analyzed based on various risk scores. Subsequently, functional enrichment analysis was performed on the differential genes identified in the risk model. Moreover, we investigated the expression of particular genes in chronic liver diseases utilizing datasets GSE135251 and GSE142530.

**Results:**

Our findings revealed hepatocyte-specific genes (ADH4, LCAT) with notable alterations during cell maturation and differentiation, leading to the development of a novel predictive signature. The analysis demonstrated the efficacy of the model in predicting outcomes, as evidenced by higher risk scores and poorer prognoses in the high-risk group. Additionally, a nomogram was devised to forecast the survival rates of patients at 1, 3, and 5 years. Our study demonstrated that the predictive model may play a role in modulating the immune microenvironment and impacting the anti-tumor immune response in hepatocellular carcinoma. The high-risk group exhibited a higher frequency of mutations and was more likely to benefit from immunotherapy as a treatment option. Additionally, we confirmed that the downregulation of hepatocyte-specific genes may indicate the progression of hepatocellular carcinoma and aid in the early diagnosis of the disease.

**Conclusion:**

Our research findings indicate that ADH4 and LCAT are genes that undergo significant changes during the differentiation of hepatocytes into cancer cells. Additionally, we have created a unique predictive signature based on genes specific to hepatocytes.

**Supplementary Information:**

The online version contains supplementary material available at 10.1186/s12920-024-01871-1.

## Introduction

Hepatocellular carcinoma (HCC) ranks as the sixth most prevalent cancer and the third leading cause of cancer-related mortality on a global scale. HCC is the fifth most significant contributor to morbidity and mortality worldwide, with a higher prevalence in males [1]. The main risk factors associated with HCC include chronic infection with hepatitis B virus (HBV) or hepatitis C virus (HCV), consumption of aflatoxin-contaminated food, alcoholism, obesity, type 2 diabetes, smoking, and non-alcoholic fatty liver disease (NAFLD). These risk factors may vary in prevalence across different geographical regions [2]. The Barcelona Clinic Liver Cancer (BCLC) staging system is currently the most widely accepted method for staging hepatocellular carcinoma. This system considers factors such as tumor burden, patient functional status, liver function, and other relevant indicators to guide the selection of clinical treatment strategies and predict prognosis [3]. As research progresses, the comprehension of hepatocellular carcinoma advances, leading to the development of numerous innovative treatment modalities. Presently, a variety of non-pharmacological treatment options are available for HCC, such as liver resection, liver transplantation, transcatheter arterial chemoembolization (TACE), ablation, and chimeric antigen receptor T cells immunotherapy [4]. However, the selection of treatment strategies is predominantly influenced by the tumor’s stage and the presence of associated complications [5]. Chemotherapy is a significant therapeutic modality for advanced HCC. Systemic chemotherapy involves the administration of drugs that circulate throughout the body via the bloodstream to impede the proliferation of tumor cells. Commonly utilized drugs in this treatment approach include 5-fluorouracil, capecitabine, and doxorubicin, among others. However, the efficacy of chemotherapy is constrained by tumor resistance and adverse toxicities [6]. Recent advancements in molecular targeted therapy and immunotherapy have demonstrated significant efficacy in treating patients with advanced HCC. The “T + A regimen” involving Atezolizumab and bevacizumab (Atezo-Bev) is currently the preferred first-line treatment for advanced HCC, offering greater benefits to patients compared to sorafenib [7]. The investigation of individualized therapy utilizing genome sequencing in patients with advanced HCC is currently underway [8]. Despite the availability of numerous treatment options for HCC, the overall survival rates remain suboptimal. Consequently, there is a critical need to delve deeper into identifying sensitive biomarkers for early detection and prognostic prediction in order to enhance the prognosis and treatment outcomes for individuals with HCC.

The liver, being a multifunctional organ, plays a crucial role in various physiological processes including biosynthesis, metabolism, and detoxification. The exposure of the liver to toxins often results in tissue damage and cell death [9], while chronic damage can also be caused by hepatotropic virus infections. Compensatory proliferation of hepatocytes is essential for the regeneration and maintenance of liver function following such damage and cell death [10]. The robust regenerative capacity of normal liver tissues is advantageous for restoring histological integrity, yet it facilitates the progression of HCC in the presence of abnormal liver conditions [11]. This transition from hepatocytes to HCC may signify a tumorigenic mechanism characterized by the loss of normal hepatocyte function and the subsequent compensatory upregulation of regeneration, inflammation, fibrosis, among other processes, ultimately promoting malignant transformation [12]. Consequently, investigating the process by which hepatocytes transform into cancer cells is essential for gaining novel insights into tumorigenesis.

Over the last ten years, high-throughput sequencing technology has significantly influenced the field of biology and altered its trajectory. While bulk RNA-seq technology has been extensively employed to analyze gene expression patterns at a population level, it is limited in its ability to capture individual variations within cells, potentially obscuring biologically significant differences [13]. The advent of single-cell RNA sequencing (scRNA-seq) has enabled researchers to investigate gene expression profiles at a cellular level. Single-cell RNA sequencing technology is a high-throughput sequencing method utilized for the examination of gene expression at the individual cell level. This technology offers the advantage of enabling a detailed analysis of gene expression in each cell, as opposed to traditional RNA sequencing methods which provide a collective analysis of gene expression in an entire tissue sample. Consequently, single-cell RNA sequencing technology allows for a more precise identification of variations between individual cells [14]. By investigating potential intratumoral genetic heterogeneity (ITGH), this technology facilitates a novel approach to exploring the mechanisms underlying tumor initiation and progression [15]. Furthermore, single-cell RNA sequencing plays a crucial role in identifying various cell types and states, thereby offering significant utility in the examination of cellular differentiation, development, metabolism, and disease. Utilizing algorithms, researchers can effectively visualize intercellular communication and reconstruct dynamic cell trajectories in relation to differentiation or cell cycle progression [16]. Some scholars contend that reconstructing cell trajectories and pseudotime order using scRNA-seq data enables the identification of dynamic alterations in gene expression during developmental processes [17]. By comparing variations in molecular features, researchers can assess the potential for tumor progression and potentially aid in the early formulation of intervention strategies [18]. Given the inadequacy of studies examining gene expression profiles of HCC at the hepatocyte level, further investigation is warranted to elucidate the correlation between alterations in hepatocyte-specific gene expression and prognosis, diagnosis and treatment. This study utilized single-cell RNA sequencing data to delineate the evolutionary path of hepatocytes transitioning into cancer cells, with the aim of elucidating alterations in gene expression profiles specific to hepatocytes throughout cellular maturation and differentiation. The study also sought to identify novel biomarkers and construct a predictive signature using bulk RNA sequencing data, with the ultimate goal of offering insights for early diagnosis, prognosis prediction, and treatment selection for patients afflicted with HCC.

## Materials and methods

### Preparation of data

The GSE115469 cohort was downloaded from the publicly available Gene Expression Omnibus (GEO) database, which includes five normal liver tissue samples. Twelve cohorts of primary hepatocellular carcinoma samples (P08, P09, P10, P11, P12, P13, P14, P15, P16, P17, P18, and P19) were obtained from the China National Gene Bank database (CNGBdb: CNP0000650), with nine of the cohorts consisting of both cancerous and adjacent tissue samples. The construction of the predictive signature utilized bulk RNA-sequencing data and corresponding clinical data from patients with hepatocellular carcinoma sourced from The Cancer Genome Atlas (TCGA) database, encompassing 374 tumor samples (TCGA-LIHC) and 50 adjacent samples. Furthermore, the validation cohort consisted of 240 tumor samples and 202 normal samples (ICGC-LIRI-JP) obtained from the International Cancer Genome Consortium (ICGC) cohort. Additionally, the GSE135631 cohort (*n* = 30) from the GEO database was utilized to confirm the expressions of hepatocyte-specific genes in tumor and adjacent samples. Finally, the cohorts GSE135251 (*n* = 216) and GSE142530 (*n* = 28) from the GEO database were acquired to investigate the expressions of hepatocyte-specific genes in chronic liver diseases.

### Single-cell data analysis

The “seurat” package was utilized to conduct quality control (QC) on normal and tumor samples separately [19]. Specifically, for normal samples, genes with a count of more than 200 and less than 6000 per cell were filtered, along with a requirement for mitochondrial gene expression to be less than 10%. In contrast, tumor samples were subjected to a filtering process where genes with a count of more than 1500 and less than 12,000 per cell were retained, with a stipulation for mitochondrial gene expression to be less than 5%. Subsequently, the samples were normalized individually using the “NormalizeData” package, and the top 2,000 highly variable genes were identified using the “FindVariableFeatures” function. The batch effect among samples was mitigated through the application of the “IntegrateData” function within the “seurat” package [20]. Utilizing the top 20 principal components and the top 2,000 variable genes, a total of 20,571 pooled single-cell data were obtained for further analysis, comprising 10,049 cells derived from normal tissue and 10,522 cells derived from tumor tissue. Following this, all genes were standardized using the “ScaleData” function, and dimensionality reduction was performed on the top 2,000 variable genes using the “RunPCA” function. The study utilized the top 30 principal components for cell clustering, employing the “FindNeighbors” and “FindClusters” functions with a resolution of 0.4 to identify 22 distinct cell clusters. Subsequently, t-distributed stochastic neighborhood embedding (t-SNE) [21] was applied for further dimension reduction using the top 30 principal components. Differential gene expression analysis within each cell cluster was conducted using the “FindAllMarks” function, with cell annotation facilitated by referencing the CellMarker database and analyzing marker genes of cell subsets in available hepatocellular carcinoma data [22] and normal liver data [23].

### InferCNV analysis

The “InferCNV” package was utilized to assess chromosomal copy number variation in single-cell data from a subset of hepatocytes, with normal hepatocytes serving as a reference set. Subsequently, the copy number variation (CNV) of HCC cells was inferred and calculated. The human genetic information was obtained from the https://data.broadinstitute.org/Trinity/CTAT/cnv/.

### Pseudotime analysis

To investigate the differentiation trajectory of hepatocytes, the “Monocle2.0” package was employed for pseudo-chronological analysis of HCC cells and normal cells. Monocle, a prominent tool for pseudotemporal analysis, utilized explicit principal graphs to characterize the data and reconstructed single-cell trajectories by embedding inversion graphs to improve the robustness and accuracy of predicted trajectories [24]. Cells were ranked according to pseudotime along the trajectory, leading to the identification of differentially expressed genes that influenced distinct differentiation trajectories at critical branch nodes. Heatmaps were generated for the genes surrounding these key nodes.

### Identification of the hepatocyte-specific genes

The “VennDiagram” package was used to obtain the intersection of the top 500 HCC prognostic genes obtained from the GEPIA2.0 website with branch-specific genes. The expression specificity of the intersecting genes in hepatocyte subsets was assessed individually to determine hepatocyte-specific prognostic genes. The “DEseq2” package was utilized to establish and filter differential gene conditions based on criteria of| log2 (FC)| >1 and FDR < 0.05. Subsequently, the “EnhancedVolcano” package was employed to confirm the differential gene status of specific hepatocyte subsets. Following this, the “survminer” package was utilized to conduct survival analysis on Alcohol Dehydrogenase 4 (ADH4), Lecithin-Cholesterol Acyltransferase (LCAT), and Complement C8 Beta Chain (C8B) using data from TCGA and ICGC datasets, demonstrating their prognostic significance in HCC. Finally, the differential expressions of these specific genes were futher investigated at protein levels between tumor and normal tissues using the Human Protein Atlas (HPA) database (http://www.proteinatlas.org/).

### Construction and evaluation of predictive signature based on hepatocyte-specific genes

The TCGA dataset was utilized as the training cohort and the ICGC dataset served as the independent validation cohort. To refine the gene selection process and enhance predictive accuracy, the “glmnet” package was employed to conduct Lasso Cox regression analysis using the training cohort [25], and the optimal genes were assessed through 10-fold cross validation. Subsequently, a multivariate Cox regression analysis was conducted to optimize the prognostic gene signature, resulting in the identification of the 2 most predictive genes. Risk scores were computed using linear combinations of specific gene expression and associated risk coefficients to construct a predictive signature. Patients were stratified into low-or high-risk categories based on the median cut-off value. Subsequently, the prognostic impact of the signature was assessed by generating survival curves for high-and low-risk groups in various cohorts, comparing gene expression patterns between the two groups using heatmaps, and plotting risk curves. To further validate the stability and accuracy of the signature, the “timeROC” package was applied to calculate the area under the curve (AUC).

Both univariate and multivariate Cox regression analyses were conducted to assess the potential of the risk score as an independent prognostic factor. The “ggalluvial” package was utilized to create an alluvial diagram illustrating the associations between various risk groups and pertinent clinical characteristics (such as clinical stage, sex, age) and survival outcomes. Additionally, the “rms” package was employed to develop a nomogram integrating multiple clinical variables for predicting the prognosis of patients with HCC. The calibration curve was utilized to assess the concordance between actual and predicted overall survival (OS) by the nomogram. Additionally, the “ggDCA” package was employed to evaluate the clinical applicability of the nomogram. Subsequently, Kaplan-Meier survival analysis was conducted to determine the OS of low-risk and high-risk groups across various clinical stages, with statistical significance set at *P* < 0.05.

### Immune infiltration analysis and immunotherapy response forecast based on the risk score

The “GSVA” and “GSEABase” packages were utilized to investigate variations in 13 immune functions and 28 immunocyte infiltrations among different risk groups within the TCGA-LIHC cohort. The Tumor Immune Dysfunction and Exclusion (TIDE) algorithm was utilized to assess the potential response to immunotherapy among HCC patients across varying risk categories [26], with the TIDE data retrieved from http://tide.dfci.harvard.edu/. Additionally, a selection of common immune checkpoint-related genes linked to immunotherapy were examined to evaluate their differential expression in distinct risk groups. Subsequently, data pertaining to an immunotherapy cohort involving cytotoxic T lymphocyte-associated antigen-4 (CTLA-4) inhibitors and programmed death-1 (PD-1) inhibitors was acquired from the https://tcia.at/ website for sensitivity analysis of different immune methods for patients with HCC in different risk groups.

**Analysis of the somatic mutation frequency, tumor mutation burden (TMB), and drug sensitivity based on the risk score**

The gene mutation data of patients with HCC was obtained from the TCGA database. The ‘maftools’ package was utilized to visualize the mutations of the top 20 genes in a waterfall plot and to compare mutation profiles among distinct risk groups [27]. Additionally, the tumor mutational burden (TMB) was calculated, and Kaplan-Meier survival analysis was conducted to assess survival disparities across risk groups. Subsequently, the “pRRophetic” package was employed to predict the half inhibitory concentration (IC50) for various drug treatments, enabling an analysis of differences in drug treatment sensitivity among different risk groups.

### Bioinformatics analysis

The Metascape website was utilized for conducting differential gene functionality analysis of branch trajectories and mapping the network. Additionally, the “clusterProfiler” package was employed for performing functional enrichment analysis of differential genes in various risk groups, encompassing Gene Ontology (GO) and Kyoto Encyclopedia of Genes and Genomics (KEGG). Subsequently, Gene Set Enrichment Analysis (GSEA) [28] was employed to compare functional disparities among different risk groups.

### Statistical analysis

Data analysis and figure plotting were carried out using R (version 4.1.1). This study utilized the Wilcoxon rank-sum test to compare differential expression between two independent samples, and the Kruskal-Wallis test to compare among multiple groups of independent samples. Survival analysis was conducted using the Kaplan-Meier method and the log-rank test, while multivariate Cox regression analysis was employed to investigate the prognostic value of the risk score and various clinical features. All statistical tests were two-sided, with a significance level set at *P* < 0.05.

## Results

### Single-cell transcriptome analysis to identify the cell types

**Fig. 1:**
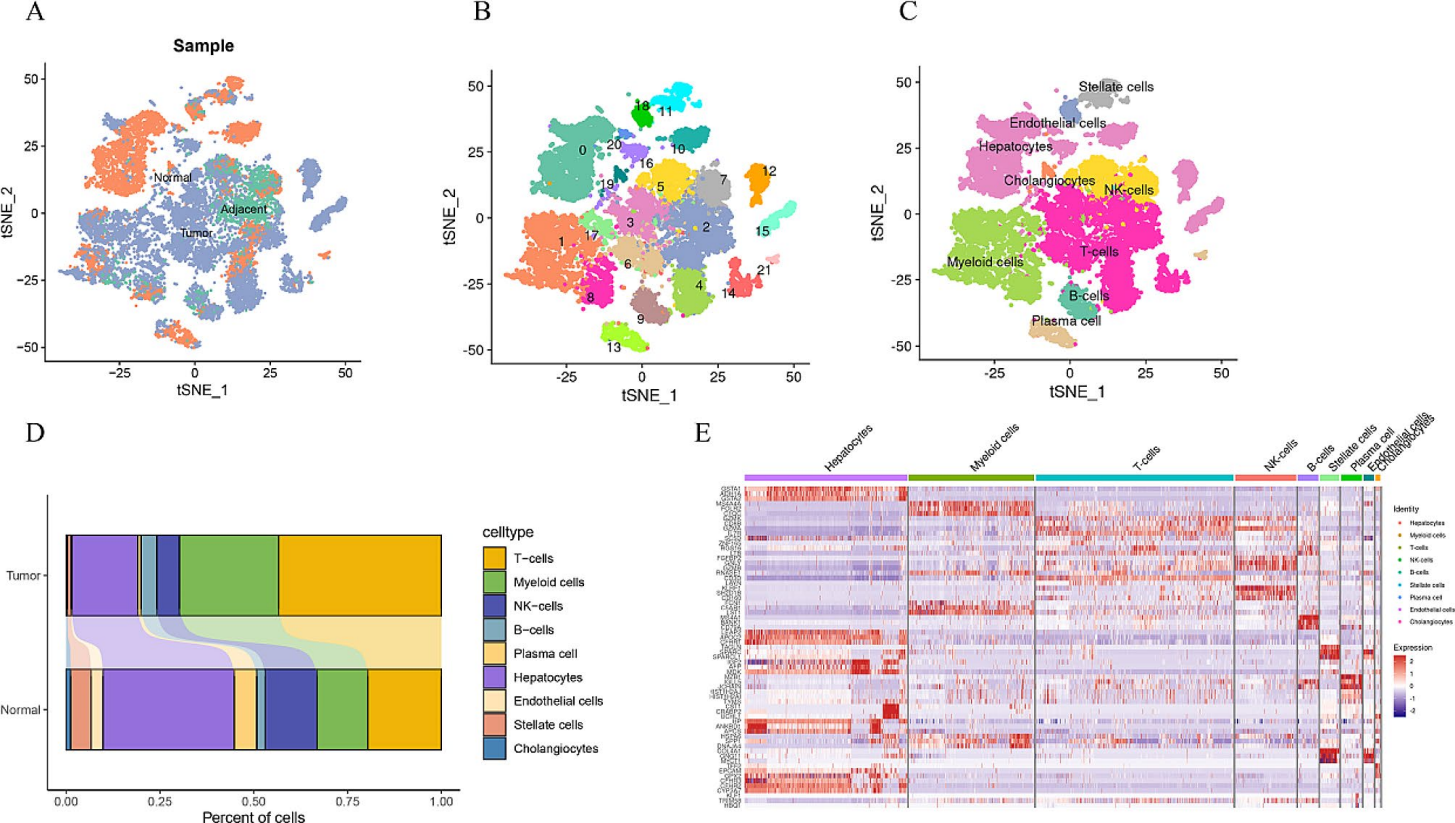
Cell-type classification of single-cell samples. (**A**) T-SNE plot is classified by sample source. (**B**) T-SNE plot of 22 cell clusters. (**C**) T-SNE plot shows cell types in single-cell samples. Cell types are annotated by the marker genes. (**D**) The proportion of cell types in tumor and normal tissues. (**E**) Heatmap of the top three marker genes in each cell cluster

This study utilized two single-cell datasets, one consisting of cancerous liver samples and the other of normal liver samples. The cancer dataset included 12 samples of HCC and 9 corresponding adjacent samples. Prior to analysis, quality control measures were implemented to exclude low-quality cells from influencing the results (Fig. S1A-C). As the sources of normal, adjacent, and HCC samples were all derived from 10X genomics analysis, we subsequently employed Seurat technology to integrate the samples. Following batch correction, the samples successfully mitigated technology-induced variation while preserving biological diversity, thereby enhancing the ability to identify distinct cell types. A total of 20,571 cells were obtained with gene expression profiles for further analysis after data integration and filtering, including 10,522 from tumor samples, 1,614 from adjacent tissues, and 8,435 from normal tissues (Fig. 1A). Principal Component Analysis (PCA) was conducted to determine data dimensions (Fig. S1D and S1E), followed by the division of cells into 22 clusters through unsupervised clustering analysis (Fig. 1B). A total of nine major cell types were identified in this study, including hepatocytes, cholangiocytes, endothelial cells, and hepatic stellate cells (HSCs), as well as various immune cells such as T cells, myeloid cells, NK cells, B cells, and plasma cell (Fig. 1C). Notably, the main cell types expressed in tumor tissues were T cells and myeloid cells, while in normal tissues, liver parenchymal cells were predominant (Fig. 1D and Fig. S1F). The top 3 highly expressed genes of each cluster were mapped on the heatmap, revealing significant differences in differential gene expression among various cell clusters of hepatocyte subsets, potentially indicating heterogeneity within these subsets (Fig. 1E).

### Identification of malignant cells and pseudotime analysis of hepatocyte subsets

**Fig. 2:**
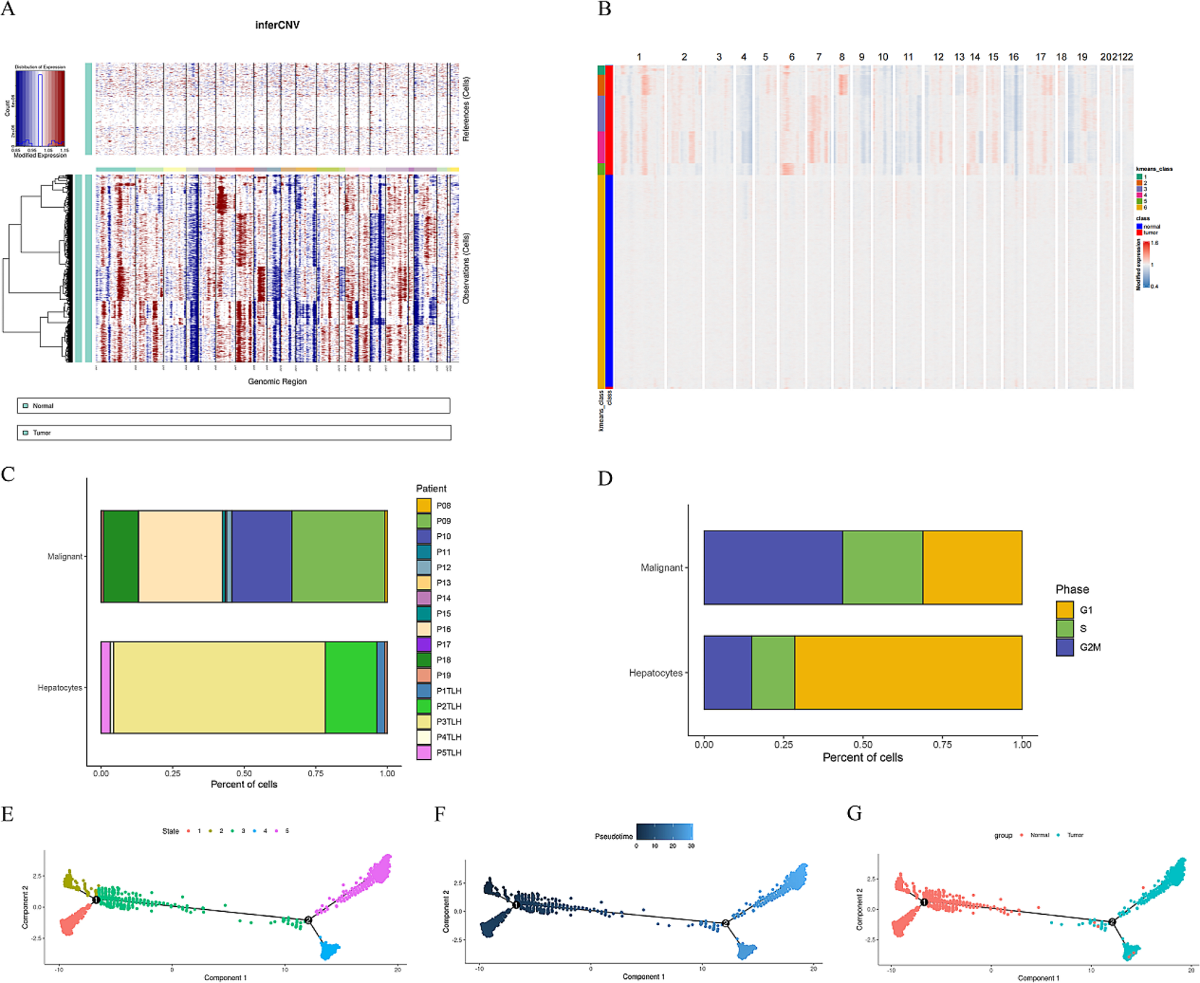
Further analysis of hepatocyte subsets. (**A**) Copy number variation in hepatocyte subsets. (**B**) Identification of chromosomal gene expression patterns. (**C**) The histogram displays the composition of liver parenchymal cells and malignant cells in the source. (**D**) The cell-cycle ratio of hepatocytes and malignant cells. The hepatocyte differentiation trajectory is shown by pseudotime analysis, and it is colored based on the cell differentiation status (**E**), the cell differentiation timing (**F**), and tissue origin (**G**)

Previous studies have suggested that hepatocarcinogenesis is initiated by the transformation of hepatocytes [29], which can occur as a result of genetic and environmental influences leading to alterations in their genetic makeup and subsequent uncontrolled cell proliferation. The alterations observed have the potential to progress towards the development of hepatocellular carcinoma by transforming hepatocytes into malignant cells. Our study involved conducting copy number variation (CNV) analysis on specific hepatocyte subsets based on their gene expression profiles within genomic regions to distinguish malignant cells. Using normal hepatocytes as the control group and malignant cells as the experimental group, we observed genome amplifications and deletions across multiple chromosomes in the malignant cells (Fig. 2A). By identifying chromosomal gene expression patterns, we were able to differentiate between hepatocytes and malignant cells within the subset of hepatocytes (Fig. 2B). A total of 3,541 normal hepatocytes and 1,821 malignant cells were utilized in the subsequent analysis following CNV correction. The composition of liver parenchymal cells and malignant cells in patient-derived samples was demonstrated through a histogram (Fig. 2C), revealing the distribution of cancer cells in liver cancer patients and hepatocytes in healthy donors, thereby mitigating potential biases in the data. Additionally, cell cycle scores were calculated to assess the cell proliferation levels across various sources (Fig. 2D). Our findings revealed that the majority of hepatocytes were found to be in the early DNA synthesis phase, while the primary malignant cells were observed in the late DNA synthesis and mitosis phases, suggesting a significant proliferation capacity. To simulate the differentiation process from normal hepatocytes to malignant cells, trajectory analysis was conducted using single-cell sequencing data. The “Monocle2” package was utilized to visualize the cell differentiation trajectory, resulting in the projection of all cells onto two roots and five branches (states) in a tree-like structure (Fig. 2E). Our research revealed a cell differentiation trajectory progressing from branch (state) 1, 2, and 3 to branch (state) 4 and 5 (Fig. 2F), with branch (state) 3 containing both normal hepatocytes and malignant cells (Fig. 2G), suggesting a potential for malignancy or varying degrees of tumor heterogeneity. As cells developed and differentiated, alterations in gene expression profiles occurred, ultimately leading to the malignant transformation of hepatocytes. Our study specifically examined differential gene expression within each branch of the root 1, as changes in these genes may influence the propensity for malignancy or predict outcomes.

### Functional analysis of differential gene expression and the identification of specific genes

**Fig. 3:**
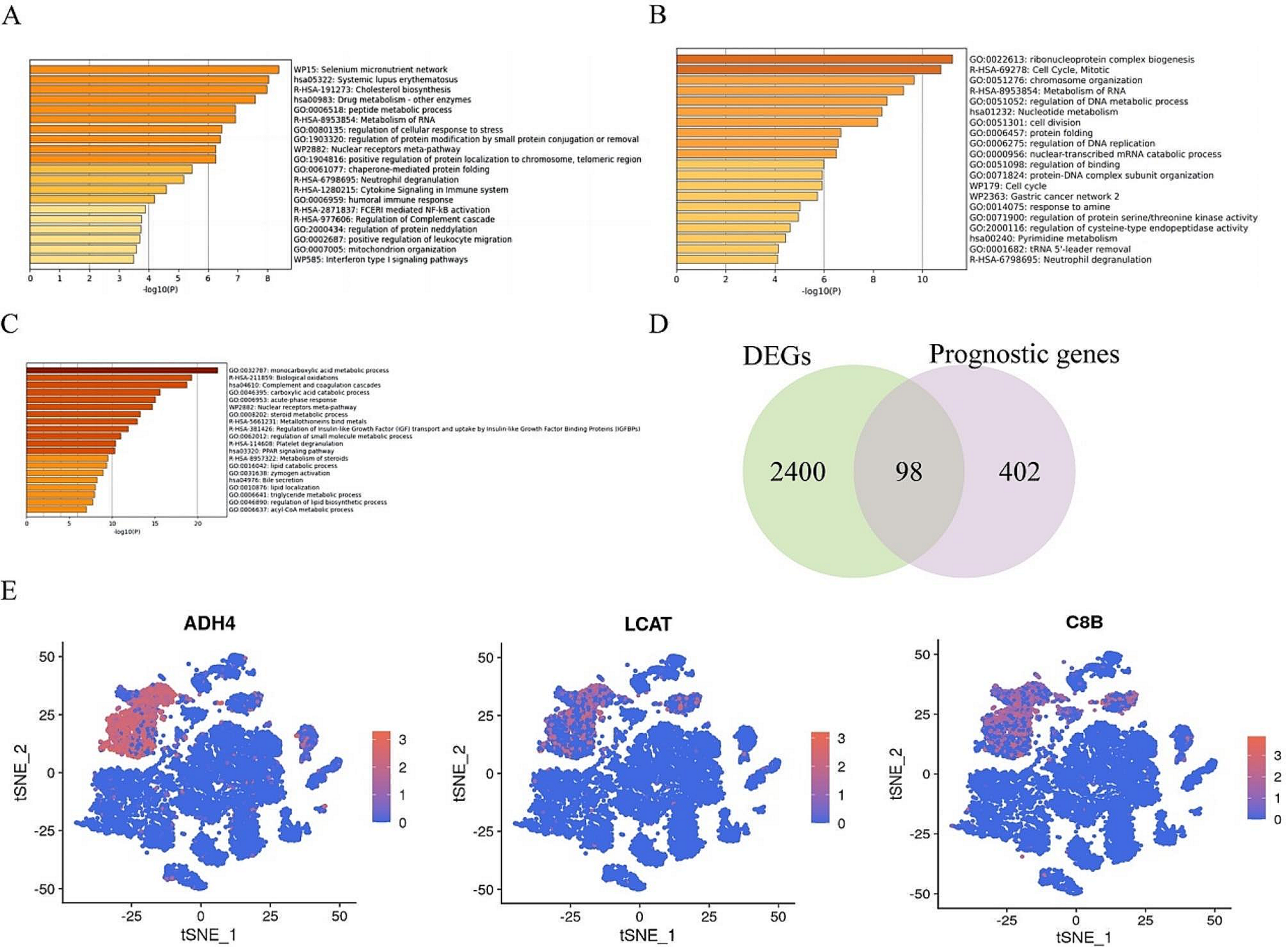
Functional analysis of differential gene expression and identification of specific genes. Genes were divided into 3 clusters according to the expression pattern of the branch heat map, and the functional analysis of the top 200 differential genes in cluster 3 (**A**), cluster 1 (**B**), and cluster 2 (**C**) were performed using the Metascape website. (**D**) Wayne diagram for the identification of differential genes. (**E**) The t-SNE plots for the distribution of specific genes

The genes were categorized into three clusters according to the expression pattern observed in the branch heatmap (Fig. S2), with clusters 1 and 3 showing high expression levels in malignant cells, while cluster 2 exhibited significantly decreased expression in malignant cells. To elucidate the functional bias and biological relevance of distinct cell populations, we chose the top 200 genes from each cluster for functional analysis using the Metascape website to investigate alterations in cellular differentiation. Our analysis revealed that cluster 3 differential genes were predominantly enriched in the selenium micronutrient network, systemic lupus erythematosus, cholesterol biosynthesis, drug metabolism, and RNA metabolism, among others (Fig. 3A). In contrast, cluster 1 differential genes showed significant enrichment in ribonucleoprotein complex biogenesis, mitotic cell cycle, chromosome organization, RNA metabolism, and regulation of DNA metabolic processes, as depicted in Fig. 3B. Additionally, cluster 2 differential genes exhibited significant enrichment in monocarboxylic acid metabolism processes, biological oxidations, complement and coagulation cascades, carboxylic acid catabolism process, and acute-phase response, as illustrated in Fig. 3C. The differential genes identified in cluster 2 were found to be associated with normal hepatocyte function, which was observed to be suppressed in cancer cells. These findings suggest that the progression of hepatocyte malignancy may be characterized by the absence of normal hepatocyte function and heightened cellular proliferation. Furthermore, the changes in genetic, molecular, and mechanistic pathways may play a significant role in the initiation and progression of HCC. Subsequently, we conducted an analysis to identify 98 differential genes with prognostic value in hepatocytes by intersecting the first 500 differential genes with prognostic value in HCC from the GEPIA2.0 website and the differentially expressed genes (DEGs) on both sides of the root point using a Venn diagram (Fig. 3D and Table S1). We individually assessed whether the intersecting genes were selectively expressed in hepatocyte subsets and pinpointed three marker genes (ADH4, LCAT, and C8B) located in cluster 2, demonstrating their specific expression in hepatocyte subsets through t-SNE plots (Fig. 3E).

### Validation of the expression and prognostic value of specific genes

**Fig. 4:**
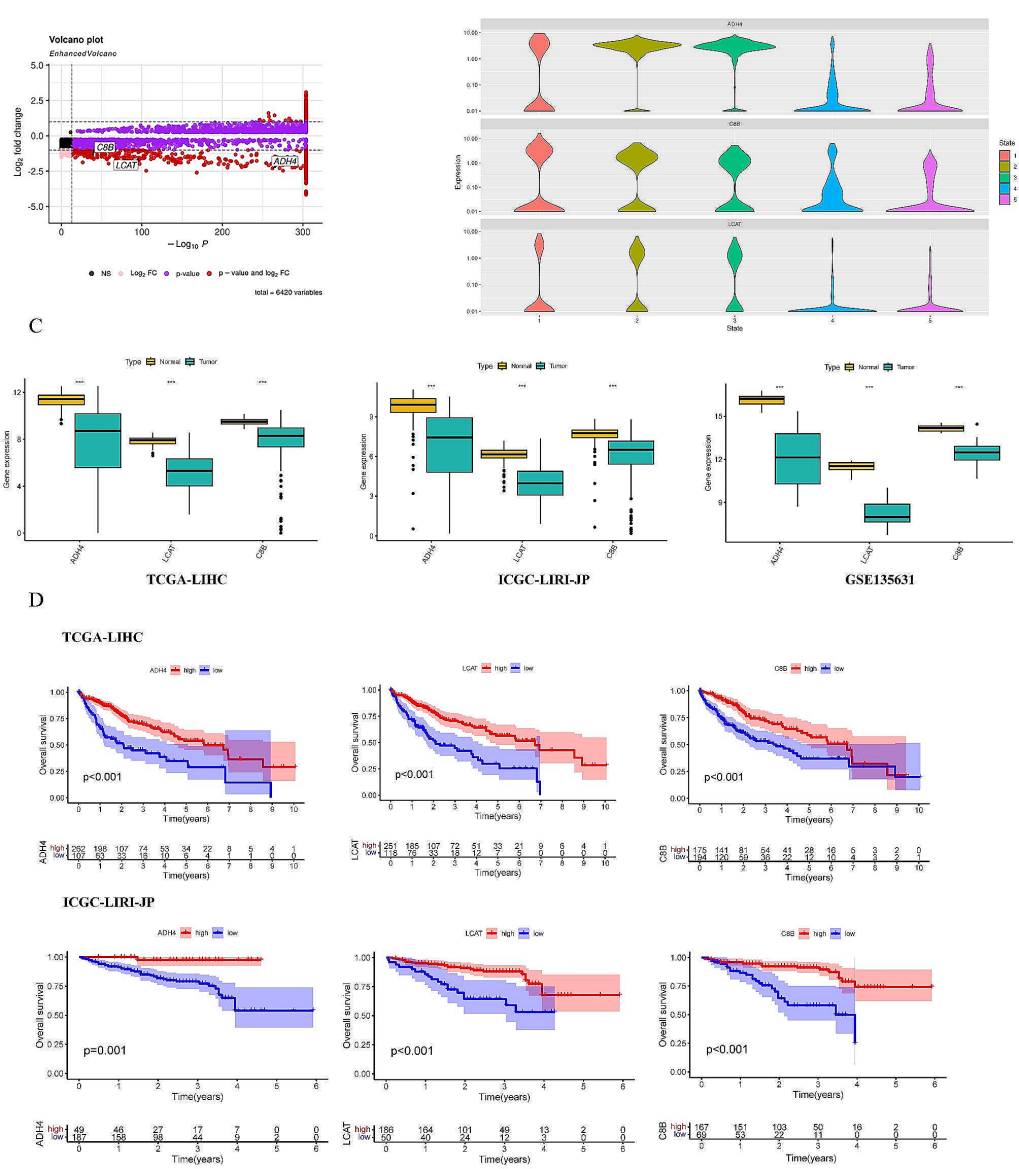
Validation of the expression and prognostic value of specific genes. (**A**) The volcano plot shows the differential expression of specific genes in hepatocytes and malignant cells. (**B**) The violin plots displays the expression changes of specific genes under pseudotime analysis. (**C**) Validation of differential expression of specific genes in normal liver and HCC tissues utilizing TCGA-LIHC, ICGC-LIRI-JP and GSE135631 cohorts. (**D**) Validation of the survival analysis by using the TCGA-LIHC and ICGC-LIRI-JP cohorts respectively. * *P* < 0.05, ** *P* < 0.01, *** *P* < 0.001, *ns*: The difference is not significant

The analysis revealed a significant reduction in the expression of three identified genes in malignant cells. Furthermore, differential expression of these genes in hepatocyte subsets was observed through the volcano plot, with notable changes in expression levels at positions of significance (Fig. 4A). Additionally, pseudotime analysis indicated a dramatic alteration in gene expression with changes in cell status, ultimately leading to a decrease in expression levels in the malignant stage (Fig. 4B). These findings suggest that the three genes undergo significant expression changes from normal hepatocytes to cancer cells, potentially playing a crucial role in tumorigenesis. Furthermore, the analysis of differential expression of the three genes in normal and tumor tissues revealed a high level of expression in normal tissues (Fig. 4C). To validate the prognostic significance of these genes, Kaplan-Meier survival curves were generated for patients from TCGA-LIHC and ICGC-LIRI-JP cohorts, demonstrating a statistically significant association between decreased gene expression and poorer prognosis (Fig. 4D). Additionally, protein expression levels of these genes were investigated based on the immunohistochemical results from the HPA database. The results showed that ADH4 and C8B expression levels were downregulated in tumor tissues compared with non-tumor tissues (Fig. S3). Unfortunately, there was a lack of data on LCAT protein expression in the HPA database.

### Identification and validation of a predictive signature based on hepatocyte-specific genes

**Fig. 5:**
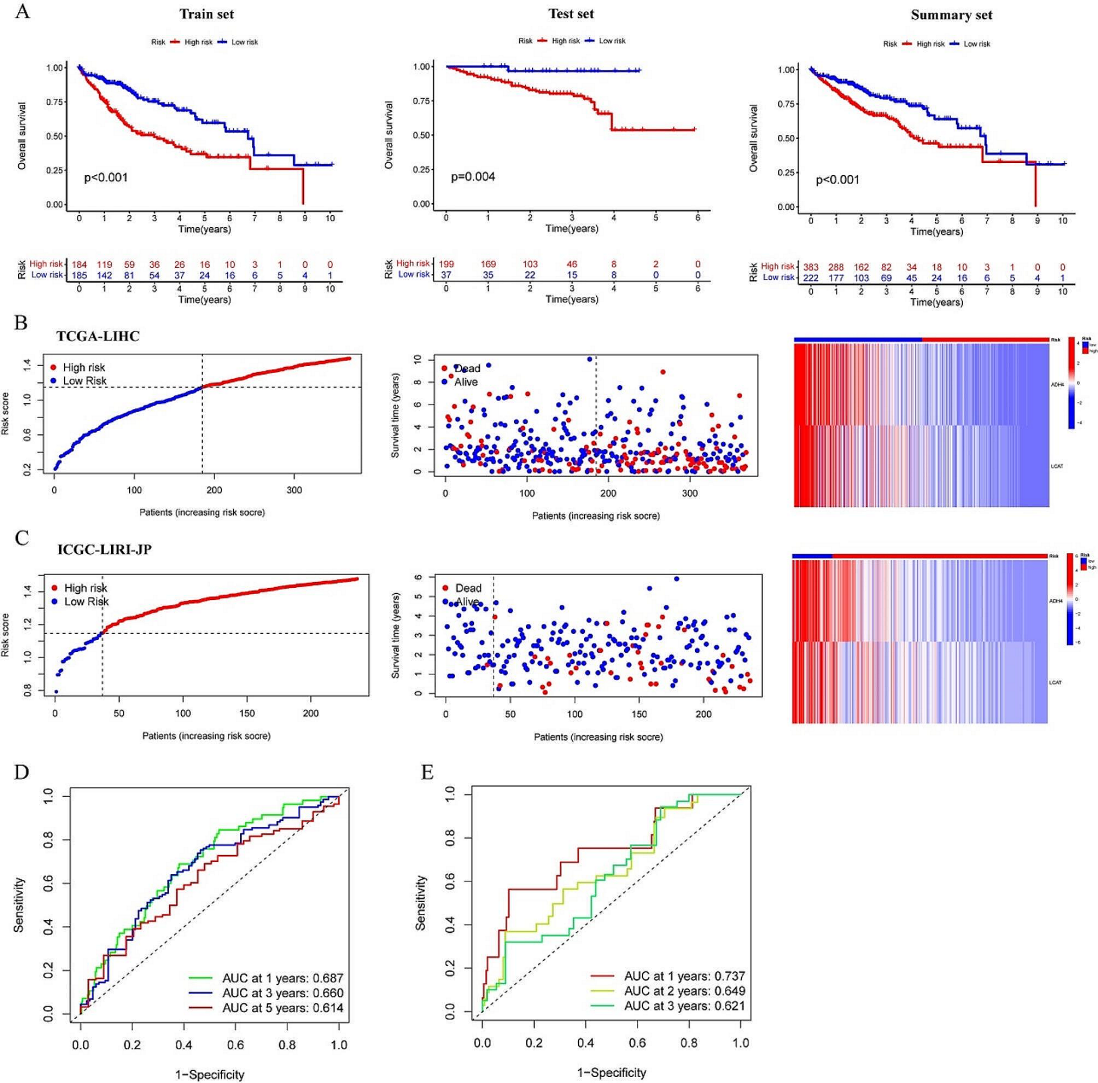
Construction and evaluation of the predictive signature. (**A**) Analysis of the overall survival of patients with HCC from TCGA, ICGC, and summary cohorts. (**B-C**) The distribution of patient’s survival and gene expression in the high-and low-risk groups was analyzed by utilizing the training cohort (TCGA-LIHC) and the validation cohort (ICGC-LIRI-JP). (**D**) The ROC curve for the training cohort (TCGA-LIHC). (**E**) The ROC curve for the validation cohort (ICGC-LIRI-JP). Differences were considered statistically significant at *P* < 0.05

The TCGA-LIHC dataset was utilized as the training cohort for the development of a predictive signature. Initially, a univariate survival analysis revealed significant associations between three genes and survival outcomes (Fig. 4D). Subsequently, Lasso regression analysis was performed for further investigation (Fig. S4A and S4B), confirming the relevance of the three genes. Finally, a gradual multivariable Cox regression analysis was conducted to refine the prognostic signature, ultimately identifying the two most predictive genes, “ADH4” and “LCAT”, and calculating the risk score of the signature. The risk score was calculated using the formula h0(t)*e(expADH4*-0.0002325 + expLCAT*-0.0028485), with a constant value of 0.398272 for lnh0(t). Patients were stratified into low- and high-risk groups based on the ranked risk scores. To evaluate the predictive performance, external validation was conducted using the ICGC-LIRI-JP cohort. The risk score was recalculated for each patient in the validation cohort using the same formula, and patients were categorized into low-and high-risk groups based on the median risk score in the training cohort. The K-M survival curve was utilized to illustrate the disparity in prognosis between high-risk and low-risk patient groups, as depicted in Fig. 5A. Analysis of both cohorts revealed a correlation between higher risk scores and increased mortality rates among HCC patients, accompanied by downregulation of HCC prognosis protective genes (ADH4, LCAT) as shown in Fig. 5B and C. To evaluate the predictive performance of the signature, the time-dependent area under the ROC curve of the predictive model was calculated. With satisfactory results, the area under the ROC curve for the 1-, 3-, and 5-year overall survival rates was found to be 0.687, 0.660, and 0.614, respectively, in the training cohort (Fig. 5D). In the validation cohort (Fig. 5E), the corresponding values for the 1-, 2-, and 3-year overall survival rates were 0.737, 0.649, and 0.621.

**Fig. 6:**
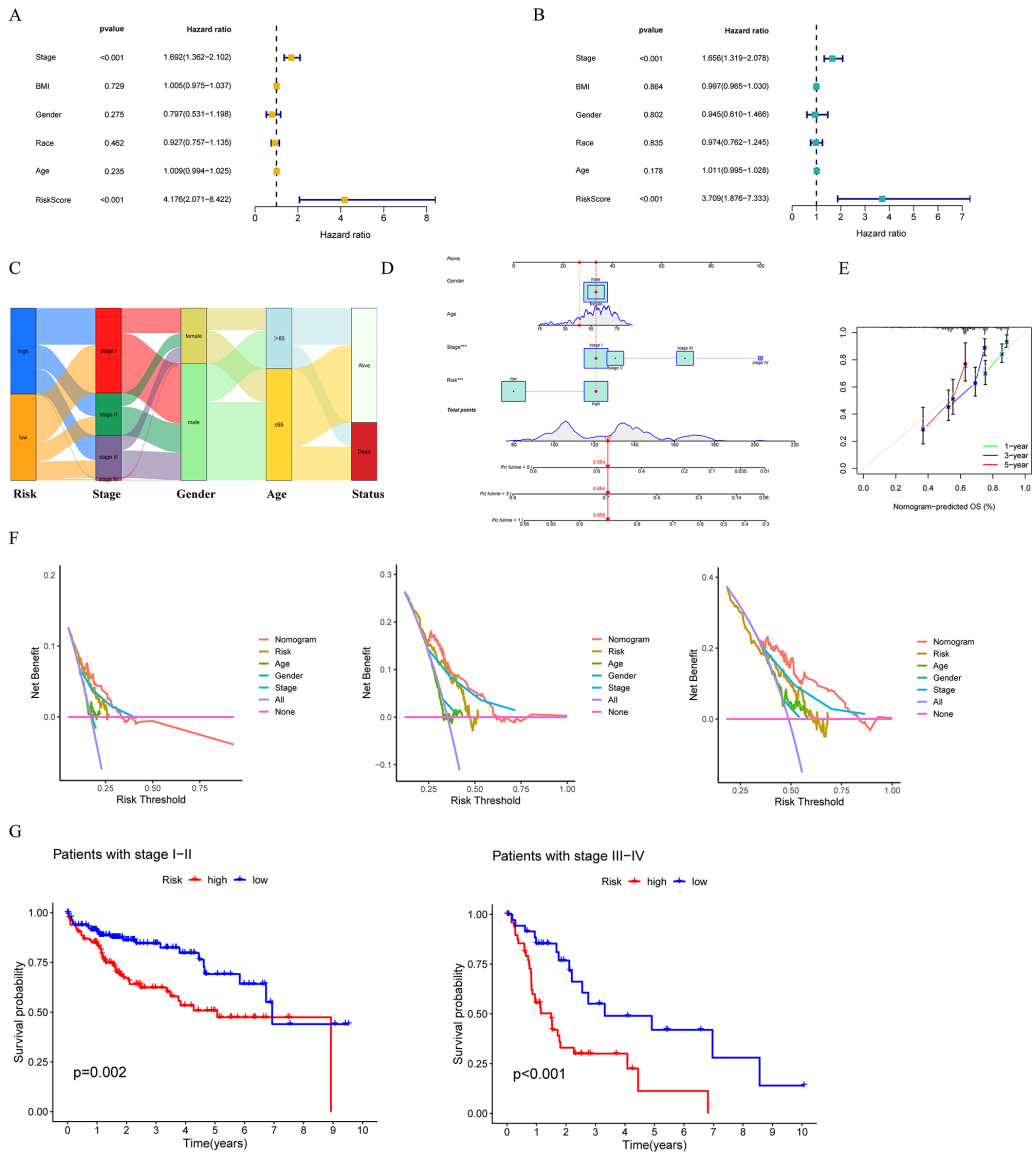
Development of a predictive signature based on specific gene expression of hepatocytes. The forest plots were established based on the univariate (**A**) and multivariate (**B**) Cox regression analysis in the TCGA-LIHC cohort. (**C**) The Mulberry plot was constructed based on risk groups, clinical stages, sex, age, and survival status. (**D**) The nomogram was established by combining risk groups with clinicopathological features in the TCGA-LIHC cohort. (**E**) The calibration curve was applied to assess the predictive accuracy of the nomogram. (**F**) Decision curve analysis of nomogram (1-, 3-, 5- year). (**G**) The K-M curves for different clinical stages were built using the TCGA-LIHC cohort. * *P* < 0.05, ** *P* < 0.01, *** *P* < 0.001, *ns*: The difference is not significant

Additionally, an analysis was conducted to assess the influence of the risk score and five clinical factors (tumor stage, body mass index, sex, race, and age) in the training cohort on prognosis, in order to determine the independent prognostic value of the two-gene predictive signature in clinical practice. In univariate Cox regression analysis on the training cohort, which displayed that the risk score and clinical stage were independent prognostic factors (Fig. 6A); multivariate Cox regression analysis indicated that the risk score and clinical stage had prognostic value independent from other clinical factors (Fig. 6B). In addition, we also performed univariate and multivariate regression analysis on the validation cohort, and the results showed that the risk score, clinical stage, and gender had prognostic value apart from other factors (Fig. S4C and S4D). In addition, a Mulberry plot was created to visually represent the relationship between various risk groups based on clinically relevant characteristics such as clinical stages, sex, and age, as well as survival status (Fig. 6C). Disparities in survival status among the different risk groups were analyzed (Fig. S4E), and it was demonstrated that the risk scores associated with varying survival statuses were statistically significant, indicating that a higher risk score was correlated with a poorer prognosis (Fig. S4F). Subsequently, a nomogram was developed utilizing risk groups and clinicopathological features within the TCGA-LIHC cohort to further forecast patient survival at 1-, 3-, and 5-year. As depicted in Fig. 6D, the survival rates at 1-, 3-, and 5-year were found to be 0.859, 0.694, and 0.554, respectively, when the total score reached 127 points. Additionally, a calibration curve was constructed to assess the predictive accuracy of the nomogram for survival (Fig. 6E). Subsequently, decision curve analysis (DCA) was performed on the nomogram model (Fig. 6F). Furthermore, a nomogram was developed based on the risk groups and clinical factors of the validation cohort, and DCA was carried out accordingly (Fig. S4G and S4H). Collectively, the establishment of the risk signature demonstrated clinical utility and potential prognostic benefits. Furthermore, survival disparities were observed among risk groups across various clinical stages, with the low-risk group exhibiting notably superior outcomes compared to the high-risk group in both early and late stages of HCC (Fig. 6G).

### Analysis of the risk score with the immune status and the immunotherapy

**Fig. 7:**
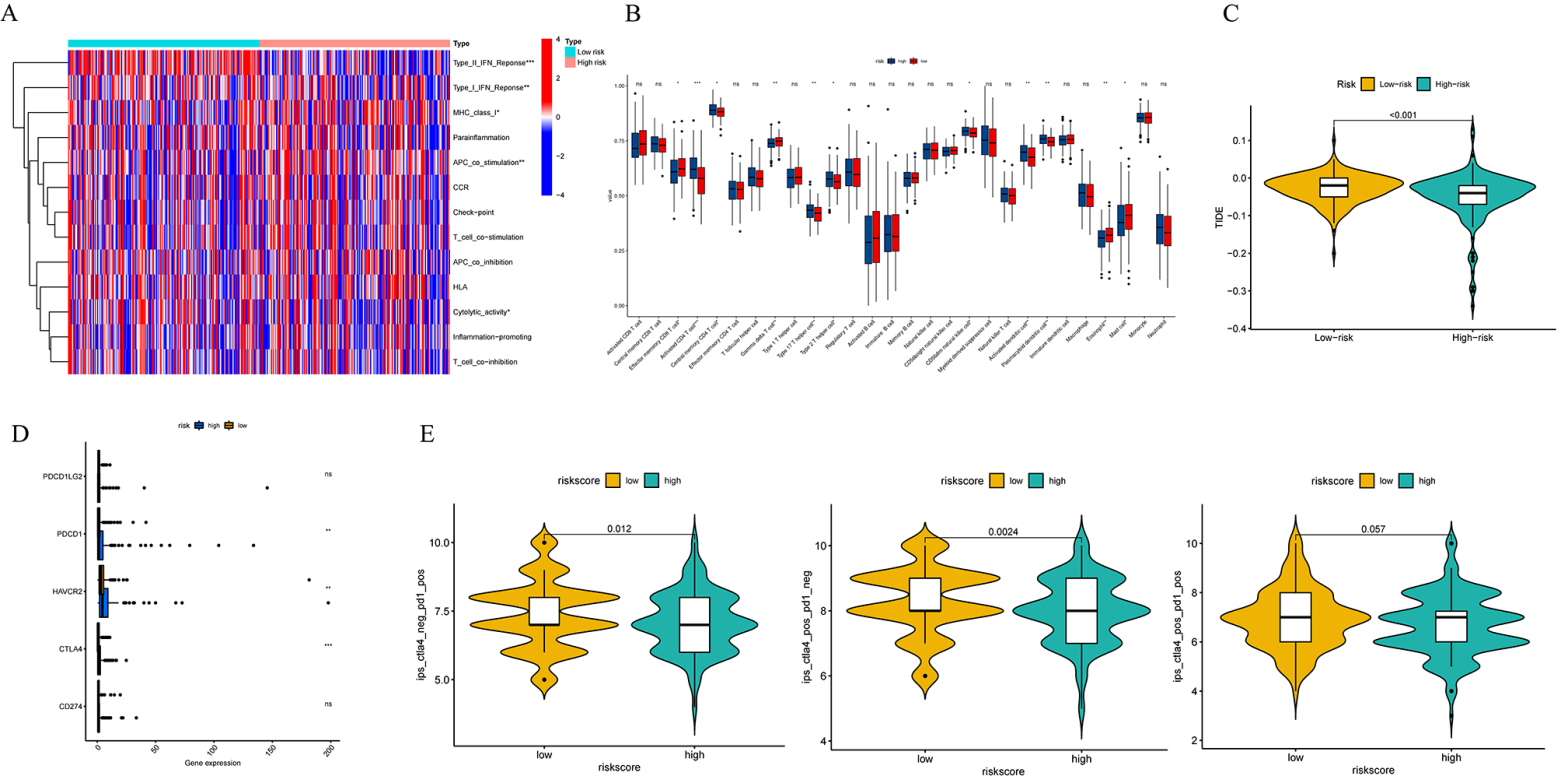
Analysis of the immune status and the immunotherapy effect according to the risk score. Immune function and immune cell expression levels between different risk groups were observed by the heatmap (**A**) and the boxplot (**B**). (**C**) Differences in the TIDE score between different risk groups. (**D**) Differences in the relevant immune checkpoint levels between different risk groups. (**E**) Differences in the immunotherapy sensitivity to the PD1 inhibitors, the CTLA-4 inhibitors, and their combination across distinct risk groups. * *P* < 0.05, ** *P* < 0.01, *** *P* < 0.001, *ns*: The difference is not significant

The single sample gene set enrichment analysis (ssGSEA) method was utilized to assess immune function and immune cell infiltration levels in different risk groups within the TCGA-LIHC cohort, and compare the differences in immune status between different risk scores. The study revealed that the low-risk group exhibited heightened immune function in cytolytic activity, type I interferon response, and type II interferon response, while displaying lower levels of APC co-stimulation and MHC-I expression (Fig. 7A). Additionally, a positive correlation was observed between the risk score and the abundance of activated CD4 + T cells, central memory CD4 + T cells, Th17, Th2, CD56bright NK cells, activated DCs, and pDCs, while an inverse correlation was found with the abundance of effector memory CD8 + T cells, γδT cells, eosinophils, and mast cells (Fig. 7B). Our hypothesis posited that the predictive signature could potentially modulate the immune microenvironment, thereby impacting the anti-tumor immune response in HCC. The observed relationship between the risk score and immune status motivated us to delve deeper into the potential role of the risk score in immunotherapy. Subsequent TIDE analysis revealed a lower TIDE score in the high-risk group (Fig. 7C). Further examination of the differential expression of common immune checkpoints across different risk groups revealed an upregulation of inhibitory checkpoints in the high-risk group, such as HAVCR2, PDCD1 and CTLA-4 (Fig. 7D). These molecules served as T cell depletion markers [30], suggesting a potential association between increased T cell depletion in the high-risk group and evasion of the anti-tumor immune response, ultimately leading to a poorer prognosis. Subsequently, we conducted a comparative analysis of immunotherapy responsiveness to PD1 inhibitors, CTLA-4 inhibitors, and their combination across distinct risk groups (Fig. 7E). Our study revealed that PD1 inhibitors or CTLA-4 inhibitors used individually demonstrated greater treatment efficacy in the high-risk group, with no significant difference observed in combination therapy. These findings suggest that patients classified in the high-risk group may derive increased benefits from immunotherapy.

### Analysis of the risk score with somatic mutation and drug treatment sensitivity

**Fig. 8:**
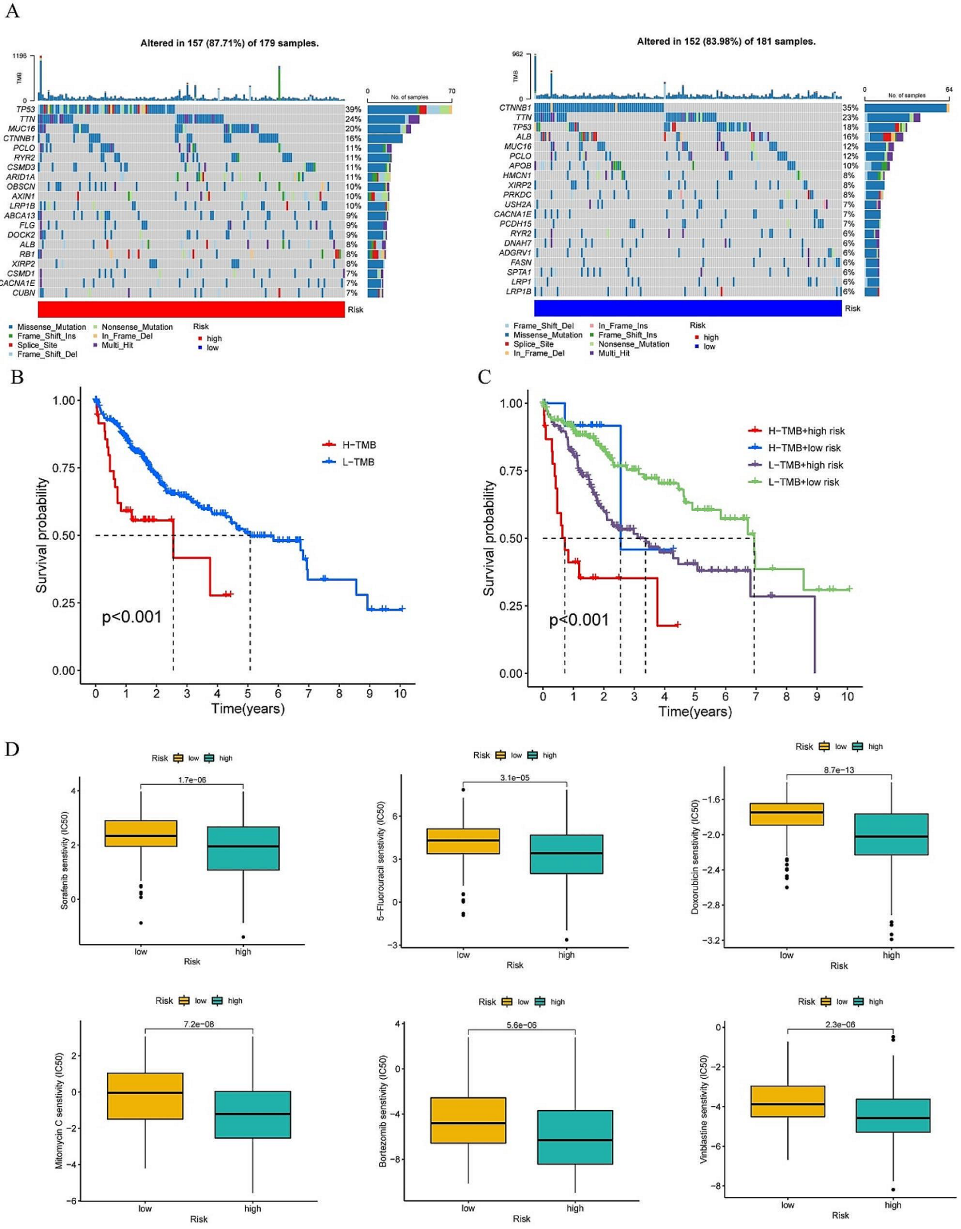
Potential role of the risk score in mutation and drug therapy. (**A**) Frequency of somatic mutation in different risk groups. (**B**) Survival analysis of the tumor mutation burden. (**C**) Subgroup survival analysis of the tumor mutation burden and the risk score. (**D**) Sensitivity analysis to chemotherapy and targeted therapy in different risk groups (including sorafenib, 5-fluorouracil, doxorubicin, mitomycin C, bortezomib, and vinblastine). The above *P* < 0.05 was considered statistically significant

The majority of cancer mutations are somatic cell mutations, with genomic instability playing a key role in the accumulation of mutations within cancer cells and the subsequent evolution of cancer genomes [31]. Somatic mutation analysis (Fig. 8A) indicated a gene mutation probability of 87.71% in the high-risk group and 83.98% in the low-risk group, suggesting that the high-risk group had more frequent mutations and may lead to adverse prognostic outcomes. Our study revealed that missense mutations were the predominant mutation type across various risk groups. Among the high-risk group, the top three genes with the highest mutation rates were TP53, TTN, and MUC16, while in the low-risk group, the top three genes were CTNNB1, TTN, and TP53. Notably, TP53 exhibited the most substantial disparity in mutation frequency between the two groups, with a significantly higher mutation rate in the high-risk group compared to the low-risk group (39% versus 18%). Subsequently, we conducted an analysis of the varying expression levels of TMB among distinct risk groups (Fig. S5). While the observed difference did not reach statistical significance, our findings indicated that individuals in the low TMB group exhibited a more favorable prognosis compared to those in the high TMB group (Fig. 8B). Intriguingly, our analysis revealed that individuals in the low-risk and low TMB group demonstrated markedly improved prognostic outcomes in comparison to those in the high-risk and high TMB group, when considering our predictive signature (Fig. 8C). Lastly, we explored the correlation between the risk score and sensitivity to drug treatment, and we explored that patients in the high-risk group had lower IC50 for several anticancer drugs, including sorafenib, 5-fluorouracil, doxorubicin, mitomycin C, bortezomib, and vinblastine (Fig. 8D), suggesting that the development of a predictive signature could aid in selecting appropriate drugs based on the clinical efficacy of anticancer agents.

### Differential enrichment analysis of the predictive signature

**Fig. 9:**
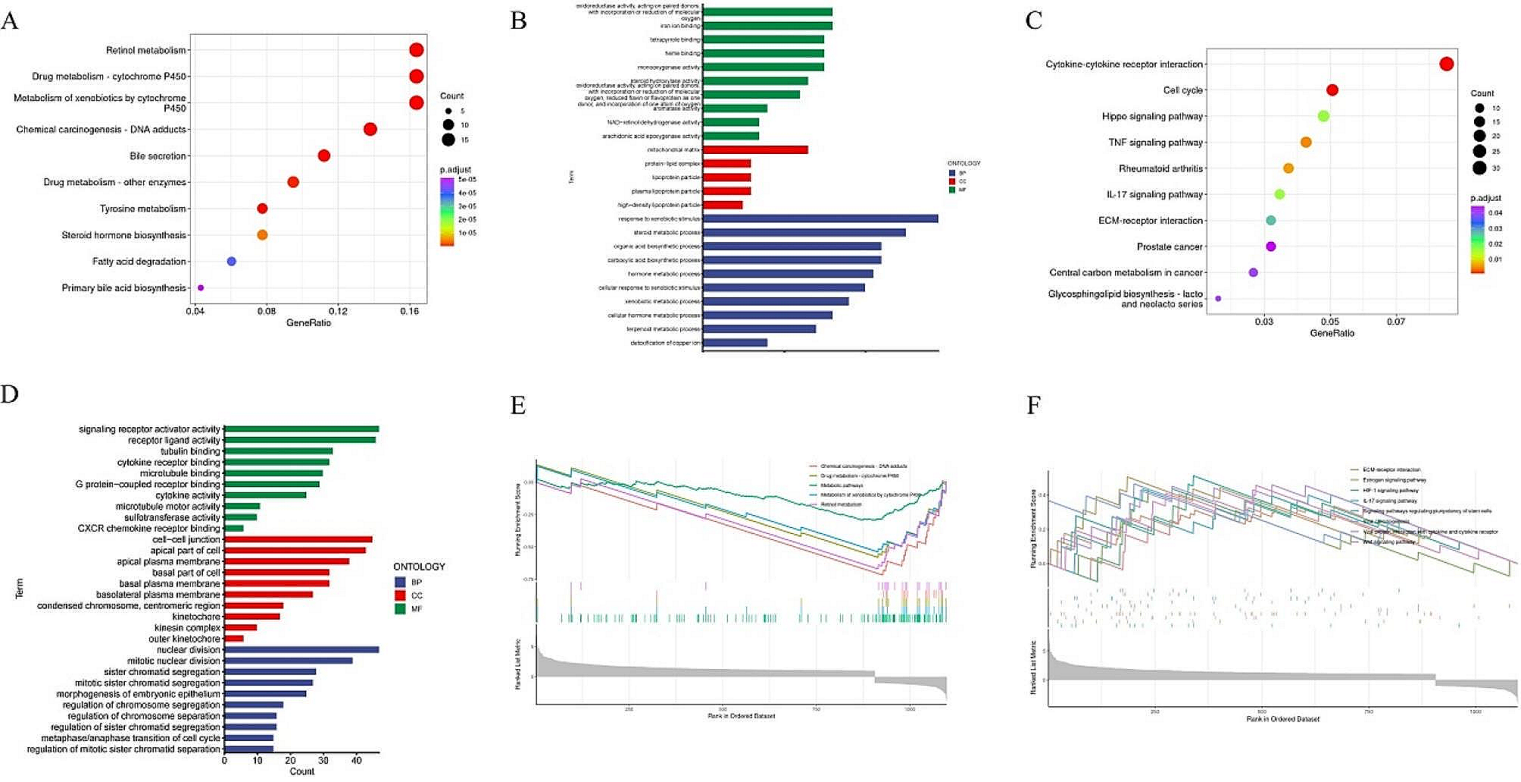
Differential enrichment analysis of the predictive signature. (**A**) KEGG enrichment analysis of differential genes in low-risk group. (**B**) GO enrichment analysis of differential genes in low-risk group. (**C**) KEGG enrichment analysis of differential genes in high-risk group. (**D**) GO enrichment analysis of differential genes in high-risk group. (**E**) GSEA enrichment analysis of differential genes in low-risk group. (**F**) GSEA enrichment analysis of differential genes in high-risk group

In order to gain further insight into the mechanisms underlying hepatocellular carcinogenesis, we conducted a differential gene enrichment analysis of the predictive signature. It was observed that the enriched pathways and functions varied significantly among the different risk groups. Specifically, the low-risk group exhibited enrichment in traditional KEGG and GO pathways related to normal hepatocyte functions, such as biosynthesis, metabolism, and detoxification (Fig. 9A and B). Conversely, the high-risk group displayed marked enrichment in pathways associated with cell division, cell proliferation, pro-inflammatory responses, and tumorigenesis (Fig. 9C and D), suggesting that gene expression in the high-risk group may have adapted to malignant characteristics. Furthermore, GSEA enrichment analysis was conducted, revealing that the low-risk group exhibited enrichment in various substance metabolic pathways and maintained normal hepatocyte metabolic functions (Fig. 9E). Conversely, the pathways enriched in the high-risk group were largely non-significant (Fig. 9F), potentially attributed to a limited number of represented genes, indicative of non-specific pathway enrichment and significant tumor heterogeneity. These findings suggest pronounced genetic heterogeneity within and between high-risk groups, resulting in the impairment of normal hepatocyte functions.

### Validation of the specificity about the decreased expression of specific genes in tumor tissues

**Fig. 10:**
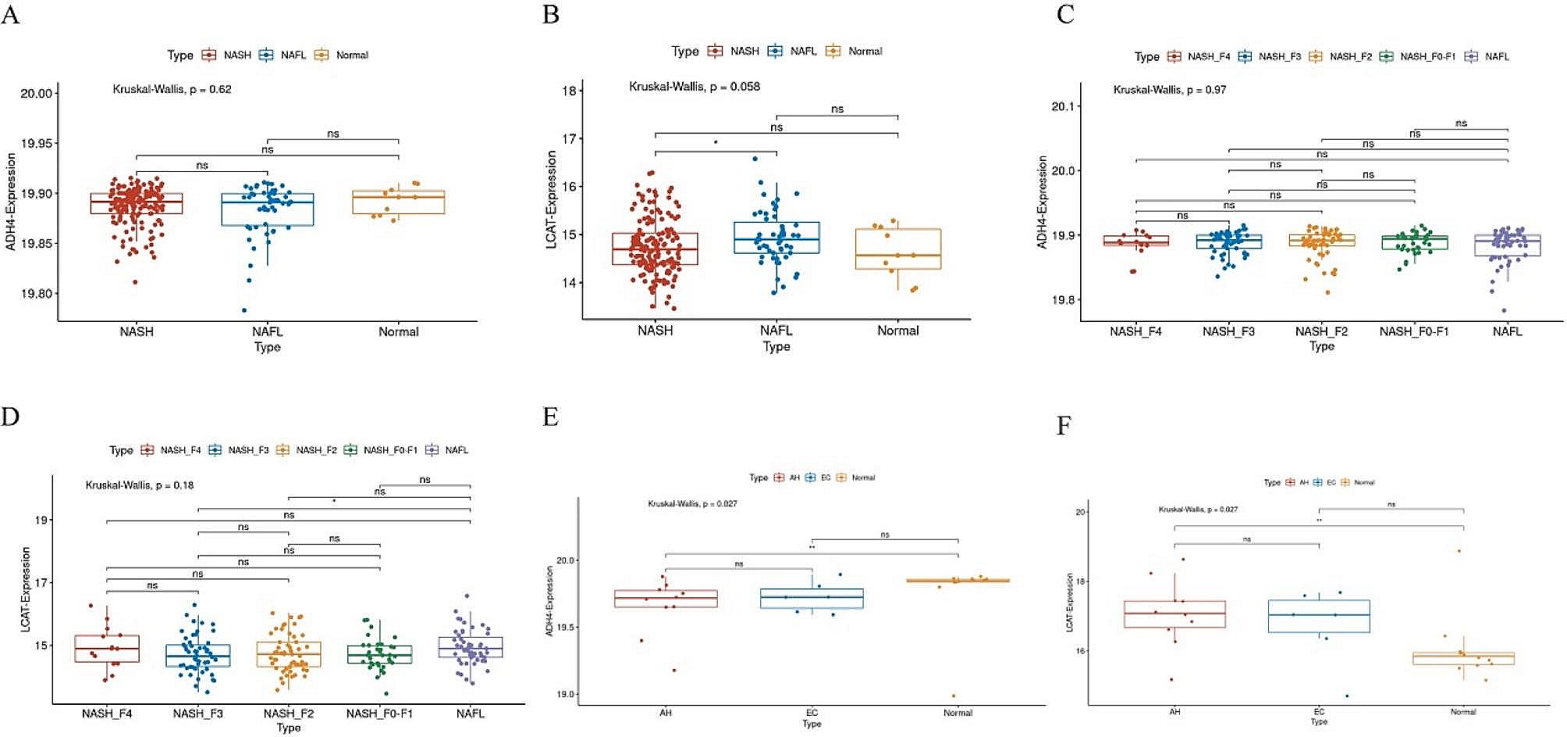
Comparison of specific gene expression in tissues from chronic liver diseases. (**A-B**) In comparison with differential expression of specific genes in patients with NASH, NAFL and controls. (**C-D**) Comparing differential expression of specific genes among different fibrosis stages in patients with NASH. (**E-F**) In comparison with differential expression of specific genes in patients with AH, alcohol-related cirrhosis (the sample name is denoted as EC) and controls. * *P* < 0.05, ** *P* < 0.01, *** *P* < 0.001, *ns*: The difference is not significant

Given the specific expression of ADH4 and LCAT in hepatocytes, it is plausible to infer that their expression levels in hepatocytes may serve as a proxy for their expression in the entirety of liver tissues. Following the validation of the prognostic significance of ADH4 and LCAT in HCC, two cohorts were incorporated to investigate whether their expression levels were selectively diminished solely in HCC. One of these cohorts encompassed individuals with non-alcoholic fatty liver disease (NAFLD) and healthy controls, with NAFLD comprising non-alcoholic steatohepatitis (NASH) and non-alcoholic fatty liver (NAFL). In this study, we conducted a comparison of gene expression levels in various disease groups and healthy controls, revealing no statistically significant differences as illustrated in Fig. 10A and B. Similar findings were observed across different stages of fibrosis in NASH as depicted in Fig. 10C and D. Furthermore, an additional cohort encompassing individuals with alcoholic hepatitis (AH), alcohol-related cirrhosis, and healthy controls was examined. Interestingly, a decrease in gene expression of ADH4 was observed in AH, while no significant difference was noted in alcohol-related cirrhosis (Fig. 10E). This finding may be attributed to the involvement of ADH4 in alcohol metabolism signaling and its potential implications, and its alcohol metabolism function would be disturbed upon alcohol exposure [32]. However, gene expression of LCAT was not found to be statistically significant in either AH or alcohol-related cirrhosis, as shown in Fig. 10F. These studies suggest that the noticeable decrease in the expression of ADH4 and LCAT in HCC may be relatively specific. In cases where patients have no history of alcohol exposure, the reduced expression of ADH4 and LCAT could potentially indicate the presence of HCC to some extent, aiding in the early diagnosis of the disease.

## Discussion

HCC represents a significant contributor to cancer-related mortality globally, presenting a substantial risk to human life and well-being. Despite the availability of numerous molecularly-targeted agents and immunotherapy strategies for HCC, a limited proportion of patients derive benefit from these treatments, largely due to the pronounced tumor heterogeneity characteristic of HCC [33]. Research has indicated that the intrinsic genetic diversity of tumor cells serves as the primary catalyst for this heterogeneity, exacerbated by the influence of the external microenvironment. This phenomenon ultimately contributes to the ineffectiveness of targeted therapy and immunotherapy, resulting in disease progression in patients with HCC [34]. The emergence of single-cell sequencing technologies represents a novel methodology for investigating tumor heterogeneity. Through scRNA-seq analysis, the differentiation between healthy and cancerous cells at various stages of tumor progression can be discerned, enabling enhanced prognostic and diagnostic capabilities through the identification of potential biomarkers and the formulation of optimal treatment strategies for cancer [35]. In this study, scRNA-seq analysis was utilized to characterize the subset of hepatocytes and elucidate their differentiation trajectory, simulating the gradual transition of hepatocytes into malignant cells. This study aimed to explore transcriptomic changes during cell differentiation by identifying hepatocyte-specific marker genes and constructing a predictive signature using a training cohort from TCGA-LIHC. The predictive value of the signature was evaluated using both the training cohort and an ICGC-LIRI-JP validation cohort, revealing that patients in the high-risk group had significantly poorer prognoses. Our study revealed higher levels of immune cell infiltration, immune checkpoints, and somatic mutation frequencies in the high-risk group, indicating a significant difference compared to the low-risk group. Additionally, integrating transcriptomic analysis of chronic liver diseases helped confirm that reduced expression of specific genes may indicate the onset of HCC and aid in early diagnosis.

Tumor heterogeneity, encompassing cancer cell and microenvironmental diversity, plays a crucial role in the development of valuable biomarkers for HCC [36]. This study identified a predictive signature comprising two specific hepatocyte genes, ADH4 and LCAT, which have been linked to the prognosis of hepatocellular carcinoma (HCC) and are considered protective factors. The alcohol dehydrogenase 4 (ADH4) gene encodes the class II alcohol dehydrogenase 4 pi subunit, a member of the alcohol dehydrogenase family associated with conditions such as alcohol dependence [37] and cancer. Previous research in HCC has shown a significant down-regulation of ADH4 mRNA and protein expression in tumor tissues, which shows a highly significant correlation with worse survival [38]. Contrarily, increased ADH4 expression has been linked to a more favorable prognosis in adenocarcinoma within non-small cell lung cancer (NSCLC) [39]. Our study additionally illustrated the heightened expression of ADH4 in hepatocytes, which was notably diminished in HCC, suggesting ADH4 may serve as an independent prognostic indicator. When compared to transcriptomic analyses of chronic liver diseases, our findings suggest that decreased ADH4 expression may have a unique predictive value for HCC, excluding cases involving alcohol exposure.

The gene encoding lecithin cholesterol acyltransferase (LCAT) produces an extracellular cholesterol esterase primarily synthesized in the liver and released into the plasma, facilitating the esterification of cholesterol and the reverse transport of cholesterol esters to the liver [40]. LCAT has been extensively investigated as a potential biomarker for cancer, with studies indicating its association with invasive breast cancer as a common serum protein marker [41] and its decreased activity in patients with colorectal cancer [42]. The study found a significant decrease in LCAT expression in tumor tissue compared to non-tumor tissue, as confirmed by immunohistochemical analysis in HCC [43]. Our research further revealed high expression of LCAT specifically in hepatocytes. Importantly, decreased LCAT expression in HCC was found to be negatively correlated with prognosis. The LCAT protein is a unique serum protein in hepatocytes, suggesting that serum levels of LCAT could serve as a convenient and useful non-invasive screening test for prediction of HCC.

In this research, a predictive signature was developed by combining two genes in the TCGA training cohort and subsequently validated using the ICGC validation cohort to assess its reliability and precision. Additionally, a nomogram was constructed based on the risk groups and clinicopathological characteristics in the TCGA-LIHC cohort to forecast the 1-, 3-, and 5-year survival rates of patients. The accuracy and practicality of the nomogram were confirmed through calibration curve and DCA, demonstrating its efficacy in predicting patient survival outcomes. Therefore, the integration of multiple factors into a nomogram demonstrated superior predictive performance compared to univariate analysis.

The tumor microenvironment harbors a diverse array of immune cells, ligand receptors, chemokines, immune checkpoints, and other molecules. The level of immunodepression has been shown to significantly impact the prognosis of HCC [44], underscoring the importance of investigating immune profiles across various risk groups. We conducted a comparative analysis of various risk groups in HCC to provide a reference for personalized treatment selection. Our analysis included factors such as immune infiltration, TIDE score, immune checkpoints, immunotherapy sensitivity, somatic mutation frequencies, TMB, and anticancer drug sensitivity. Our analysis of somatic mutation frequencies revealed TP53 mutation as the most prominent difference between high- and low-risk groups. Our findings align with existing research showing that TP53 mutation, the most prevalent mutation in cancer, is associated with increased tumor aggressiveness and poorer prognosis, particularly in HCC patients [45]. Compared to existing studies on HCC prognostic models utilizing bulk RNA-seq technology, such as those based on pyroptosis-related gene models [46] and immune infiltration-related gene models [47], there is a notable gap in research at the single-cell level. In contrast to the two current prognostic models incorporating HCC scRNA-seq data [48–49], our developed predictive model focuses on identifying hepatocyte-specific genes with significant expression changes from a cellular evolution perspective. This model demonstrates higher specificity for hepatocytes and HCC. In a word, our study investigated alterations in gene expression profiles and tumor heterogeneity during the progression of hepatocellular carcinoma, offering insights for identifying precise biomarkers and targeted therapies.

In the research, We established a predictive signature with high accuracy, prompting further investigation into its mechanisms in HCC. Based on the findings of KEGG enrichment analysis, it was observed that the high-risk group exhibited significant enrichment of differential genes primarily associated with pathways related to cell proliferation (cell cycle), pro-inflammatory processes (cytokine-cytokine receptor interaction, TNF signaling pathway), and pro-tumor mechanisms (Hippo signal pathway, IL-17 signaling pathway) [50–52]. Conversely, the low-risk group displayed enrichment of differential genes predominantly linked to essential liver function pathways (retinol metabolism, drug metabolism-cytochrome P450, bile secretion, etc.). The results of the GO enrichment analysis revealed that genes exhibiting differential expression in the high-risk group were significantly enriched in biological processes (BP) such as mitotic nuclear division and mitotic sister chromatid separation; in cellular components (CC) including the basal plasma membrane and basal part of cell; and in molecular functions (MF) such as cytokine receptor binding, signaling receptor activator activity, and receptor ligand activity. Conversely, genes showing differential expression in the low-risk group were predominantly enriched in xenobiotic metabolic processes, response to xenobiotic stimulus, and steroid metabolic process and other aspects in BP, plasma lipoprotein particle and lipoprotein particle in CC, and steroid hydroxylase and oxidoreductase activity in MF. Therefore, our findings suggest a strong correlation between the active cell proliferation, cell division, pro-inflammatory, and tumor-promoting mechanisms in the high-risk group and its unfavorable prognosis. Additionally, the hepatocytes in the high-risk group displayed impaired functionality, heightened heterogeneity, and a propensity for malignant progression. Our study further demonstrates a high degree of congruence between the predictive signature group and the simulated hepatocyte differentiation trajectory in terms of specific gene expression patterns and alterations in cellular function.

Despite the valuable predictive signature that was established, there were limitations that must be addressed. Firstly, all studies conducted were retrospective, necessitating a significant number of prospective studies for validation. Secondly, the verification of gene expression in the signature was limited to the mRNA level, highlighting the need for further validation of protein expression in hepatocytes and exploration of the molecular mechanisms underlying carcinogenesis. Future research will focus on elucidating the potential mechanisms linking the expression of specific genes in hepatocytes to the prognosis and progression of HCC.

## Conclusion

Throughout the process of hepatocyte transformation into HCC cells, notable alterations in specific genes, namely ADH4 and LCAT, were observed and found to be associated with the prognosis of HCC. The downregulation of ADH4 and LCAT expression levels may serve as potential indicators for the onset of HCC, thus facilitating early detection of the disease. Our innovative predictive signature, derived from hepatocyte-specific genes, offers a novel framework for early diagnosis, prognostic assessment, and personalized therapeutic interventions for individuals afflicted with HCC.

### Electronic supplementary material

Below is the link to the electronic supplementary material.

Supplementary Material 1

Supplementary Material 2

## Data Availability

The data used to support the results of this study were available from public databases, including TCGA (TCGA-LIHC cohort) database (https://portal.gdc.cancer.gov/), ICGC (ICGC-LIRI-JP cohort) database (https://dcc.icgc.org/), GEO (bulk RNA-sequencing data: GSE135631, GSE135251 and GSE142530. Single cell omic data: GSE115469) database (https://www.ncbi.nlm.nih.gov/geo/), China National Gene Bank (CNP0000650) database (https://db.cngb.org/). The human genetic information was obtained from the https://data.broadinstitute.org/Trinity/CTAT/cnv/. The TIDE data retrieved from http://tide.dfci.harvard.edu/. The data pertaining to an immunotherapy cohort involving CTLA-4 inhibitors and PD-1 inhibitors was acquired from the https://tcia.at/.

## Abbreviations

HCC: Hepatocellular carcinoma
scRNA-seq: single cell RNA sequencing
TCGA: The Cancer Genome Atlas
ICGC: International Cancer Genome Consortium
TIDE: Tumor immune dysfunction and exclusion
TMB: Tumor mutational burden
HBV: Hepatitis B virus
HCV: Hepatitis C virus
NAFLD: Non-alcoholic fatty liver disease
NASH: Non-alcoholic steatohepatitis
BCLC: Barcelona Clinic Liver Cancer
TACE: Transcatheter arterial chemoembolization
Bulk RNA-seq: bulk RNA sequencing
ITGH: Intratumoral genetic heterogeneity
GEO: Gene Expression Omnibus
CNGBdb: China National Gene Bank database
LIHC: Liver hepatocellular carcinoma
t-SNE: t-distributed stochastic neighbor embedding
CNV: Copy number variation
ADH4: Alcohol Dehydrogenase 4
LCAT: Lecithin-Cholesterol Acyltransferase
C8B: Complement C8 Beta Chain
HPA: The Human Protein Atlas
AUC: Area Under the ROC
OS: Overall survival
CTLA-4: Cytotoxic T lymphocyte-associated antigen-4
PD-1: Programmed death-1
IC50: Half maximal inhibitory concentration
GO: Gene Ontology
KEGG: Kyoto Encyclopedia of Genes and Genomes
GSEA: Gene Set Enrichment Analysis
PCA: Principal component analysis
HSCs: Hepatic Stellate Cells
DEGs: Differentially expressed genes
DCA: Decision curve analysis
ssGSEA: single sample gene set enrichment analysis
APC: Antigen presenting cell
NAFL: Non-alcoholic fatty liver
AH: Alcoholic hepatitis
NSCLC: Non-small cell lung cancer
BP: Biological process
CC: Cellular components
MF: Molecular function

## References

[1] Sung H, Ferlay J, Siegel RL (2021). Global Cancer statistics 2020: GLOBOCAN estimates of incidence and Mortality Worldwide for 36 cancers in 185 Countries[J]. CA Cancer J Clin.

[2] Thomas London W, Petrick JL, McGlynn KA. Cancer Epidemiology and Prevention[J]. 4th ed. Oxford University Press; 2018. pp. 635–60.

[3] Reig M, Forner A, Rimola J (2022). BCLC strategy for prognosis prediction and treatment recommendation: the 2022 update[J]. J Hepatol.

[4] Chen Z, Xie H, Hu M (2020). Recent progress in treatment of hepatocellular carcinoma[J]. Am J Cancer Res.

[5] Sangro B, Sarobe P, Hervás-Stubbs S (2021). Advances in immunotherapy for hepatocellular carcinoma[J]. Nat Rev Gastroenterol Hepatol.

[6] Ikeda M, Morizane C, Ueno M (2018). Chemotherapy for hepatocellular carcinoma: current status and future perspectives[J]. Jpn J Clin Oncol.

[7] Foerster F, Galle PR (2021). The current Landscape of clinical trials for systemic treatment of HCC[J]. Cancers (Basel).

[8] Ikeda S, Lim JS, Kurzrock R (2018). Analysis of tissue and circulating Tumor DNA by Next-Generation sequencing of Hepatocellular Carcinoma: implications for targeted Therapeutics[J]. Mol Cancer Ther.

[9] Chembazhi UV, Bangru S (2021). Cellular plasticity balances the metabolic and proliferation dynamics of a regenerating liver[J]. Genome Res.

[10] Font-Burgada J, Shalapour S, Ramaswamy S (2015). Hybrid Periportal Hepatocytes regenerate the injured liver without giving rise to Cancer[J]. Cell.

[11] Michalopoulos GK, Bhushan B (2021). Liver regeneration: biological and pathological mechanisms and implications[J]. Nat Rev Gastroenterol Hepatol.

[12] Luedde T, Kaplowitz N, Schwabe RF (2014). Cell death and cell death responses in liver disease: mechanisms and clinical relevance[J]. Gastroenterology.

[13] Olsen TK, Baryawno N (2018). Introduction to single-cell RNA Sequencing[J]. Curr Protoc Mol Biol.

[14] Slovin S, Carissimo A, Panariello F (2021). Single-cell RNA sequencing analysis: a step-by-step Overview[J]. Methods Mol Biol.

[15] Chen G, Ning B, Shi T, Single-Cell RNA-S. Front Genet. 2019;10:317. Technologies and Related Computational Data Analysis[J]. 10.3389/fgene.2019.00317 PMC6460256 31024627

[16] Hwang B, Lee JH, Bang D (2018). Single-cell RNA sequencing technologies and bioinformatics pipelines[J]. Exp Mol Med.

[17] Griffiths JA, Scialdone A, Marioni JC (2018). Using single-cell genomics to understand developmental processes and cell fate decisions[J]. Mol Syst Biol.

[18] Baslan T, Hicks J (2017). Unravelling biology and shifting paradigms in cancer with single-cell sequencing[J]. Nat Rev Cancer.

[19] Butler A, Hoffman P, Smibert P (2018). Integrating single-cell transcriptomic data across different conditions, technologies, and species[J]. Nat Biotechnol.

[20] Tran HTN, Ang KS, Chevrier M (2020). A benchmark of batch-effect correction methods for single-cell RNA sequencing data[J]. Genome Biol.

[21] Kobak D, Berens P (2019). The art of using t-SNE for single-cell transcriptomics[J]. Nat Commun.

[22] Sun Y, Wu L, Zhong Y, Zhou K (2021). Single-cell landscape of the ecosystem in early-relapse hepatocellular carcinoma[J]. Cell.

[23] MacParland SA, Liu JC, Ma XZ (2018). Single cell RNA sequencing of human liver reveals distinct intrahepatic macrophage populations[J]. Nat Commun.

[24] Qiu X, Mao Q, Tang Y (2017). Reversed graph embedding resolves complex single-cell trajectories[J]. Nat Methods.

[25] Tibshirani R (1997). The lasso method for variable selection in the Cox model[J]. Stat Med.

[26] Jiang P, Gu S, Pan D (2018). Signatures of T cell dysfunction and exclusion predict cancer immunotherapy response[J]. Nat Med.

[27] Mayakonda A, Lin DC, Assenov Y (2018). Maftools: efficient and comprehensive analysis of somatic variants in cancer[J]. Genome Res.

[28] Subramanian A, Tamayo P, Mootha V et al. Gene set enrichment analysis: A knowledge-based approach for interpreting genome-wide expression profiles[J]. Proc. Natl. Acad. Sci. USA,2005, 102:15545–15550. 10.1073/pnas.0506580102 PMC1239896 16199517

[29] Yamamoto M, Xin B, Nishikawa Y. Mouse Model for Hepatocellular Carcinoma and Cholangiocarcinoma Originated from Mature Hepatocytes[J]. Methods Mol Biol, 2019, 1905:221–236. 10.1007/978-1-4939-8961-4_20 30536104

[30] Ando M, Ito M, Srirat T (2020). Memory T cell, exhaustion, and tumor immunity[J]. Immunol Med.

[31] Yi S, Lin S, Li Y (2017). Functional variomics and network perturbation: connecting genotype to phenotype in cancer[J]. Nat Rev Genet.

[32] Luo J, Hou Y, Ma W (2021). A novel mechanism underlying alcohol dehydrogenase expression: hsa-miR-148a-3p promotes ADH4 expression via an AGO1-dependent manner in control and ethanol-exposed hepatic cells[J]. Biochem Pharmacol.

[33] Craig AJ, von Felden J, Garcia-Lezana T (2020). Tumour evolution in hepatocellular carcinoma[J]. Nat Rev Gastroenterol Hepatol.

[34] Qin R, Zhao H, He Q (2022). Advances in single-cell sequencing technology in the field of hepatocellular carcinoma[J]. Front Genet.

[35] Levitin HM, Yuan J, Sims PA (2018). Single-cell transcriptomic analysis of Tumor Heterogeneity[J]. Trends Cancer.

[36] Zhang Q, Lou Y, Yang J (2019). Integrated multiomic analysis reveals comprehensive tumour heterogeneity and novel immunophenotypic classification in hepatocellular carcinomas[J]. Gut.

[37] Chowdhury NP, Moon J, Müller V (2021). Adh4, an alcohol dehydrogenase controls alcohol formation within bacterial microcompartments in the acetogenic bacterium Acetobacterium woodii[J]. Environ Microbiol.

[38] Wei RR, Zhang MY, Rao HL (2012). Identification of ADH4 as a novel and potential prognostic marker in hepatocellular carcinoma[J]. Med Oncol.

[39] Wang P, Zhang L, Huang C (2018). Distinct prognostic values of Alcohol Dehydrogenase Family members for Non-small Cell Lung Cancer[J]. Med Sci Monit.

[40] Norum KR (2017). The function of lecithin:cholesterol acyltransferase (LCAT)[J]. Scand J Clin Lab Invest.

[41] Park HM, Kim H, Kim DW (2020). Common plasma protein marker LCAT in aggressive human breast cancer and canine mammary tumor[J]. BMB Rep.

[42] Mihajlovic M, Gojkovic T, Vladimirov S (2019). Changes in lecithin: cholesterol acyltransferase, cholesteryl ester transfer protein and paraoxonase-1 activities in patients with colorectal cancer[J]. Clin Biochem.

[43] Zheng Y, Liu Y, Zhao S (2018). Large-scale analysis reveals a novel risk score to predict overall survival in hepatocellular carcinoma[J]. Cancer Manag Res.

[44] Fu Y, Liu S, Zeng S (2019). From bench to bed: the tumor immune microenvironment and current immunotherapeutic strategies for hepatocellular carcinoma[J]. J Exp Clin Cancer Res.

[45] Xiao J, Liu T, Liu Z (2022). A differentiation-related gene Prognostic Index contributes to prognosis and immunotherapy evaluation in patients with Hepatocellular Carcinoma[J]. Cells.

[46] Duan S, Gao J, Lou W (2022). Prognostic signature for hepatocellular carcinoma based on 4 pyroptosis-related genes. BMC Med Genomics[J].

[47] Dai K, Liu C, Guan G (2022). Identification of immune infiltration-related genes as prognostic indicators for hepatocellular carcinoma[J]. BMC Cancer.

[48] Wang H, Yu S, Cai Q (2021). The Prognostic Model based on Tumor Cell Evolution Trajectory reveals a different Risk Group of Hepatocellular Carcinoma[J]. Front Cell Dev Biol.

[49] Li X, Wang L, Wang L (2021). Single-cell sequencing of Hepatocellular Carcinoma reveals cell interactions and cell heterogeneity in the Microenvironment[J]. Int J Gen Med.

[50] Turner MD, Nedjai B, Hurst T (2014). Cytokines and chemokines: at the crossroads of cell signalling and inflammatory disease[J]. Biochim Biophys Acta.

[51] Kowalczyk W, Romanelli L, Atkins M (2022). Hippo signaling instructs ectopic but not normal organ growth[J]. Science.

[52] Li J, Zeng M, Yan K (2020). IL-17 promotes hepatocellular carcinoma through inhibiting apoptosis induced by IFN-γ[J]. Biochem Biophys Res Commun.

