# Supplementary material for: Identification and validation of a novel predictive signature based on hepatocyte-specific genes in hepatocellular carcinoma by integrated analysis of single-cell and bulk RNA sequencing
Source: BMC Med Genomics. 2024 Apr 23;17:103. doi: 10.1186/s12920-024-01871-1 (PMC11040759; doi:10.1186/s12920-024-01871-1)
Supplement: Supplementary file 1 — Supplementary Material 1 [file 12920_2024_1871_MOESM1_ESM.docx]

**Fig. S1**

**
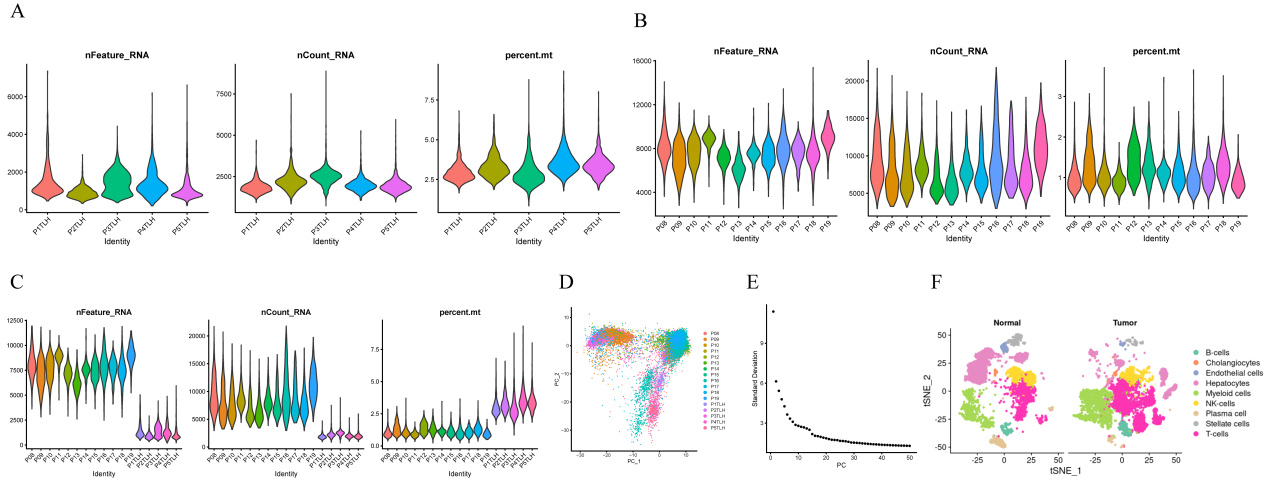
**

**Fig. S1** (**A**) The violin plot displays the indicators before quality control in the GSE115469 cohort. (**B**) The violin plot displays the indicators before quality control in the CNP0000650 cohort. (**C**) The violin plot shows the indicators after quality control in the merged data. (**D**) The plot of principal component analysis in the merged data. (**E**) The scree plot determines the choice of data dimensions. (**F**) The t-SNE plot shows cell types in tumor and normal tissues

**Fig. S2**

**
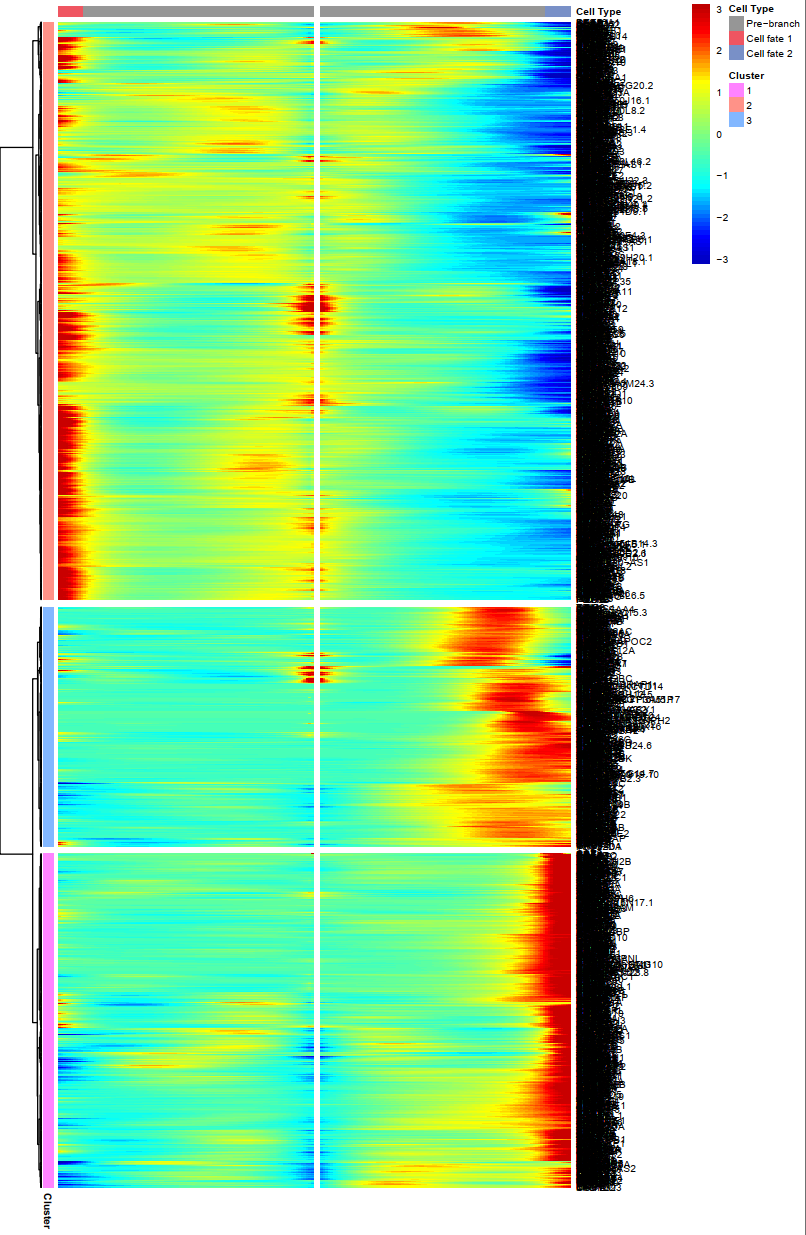
**

**Fig. S2** The heatmap demonstrates the dynamic changes of gene expression along the pseudotime

**Fig. S3**

**
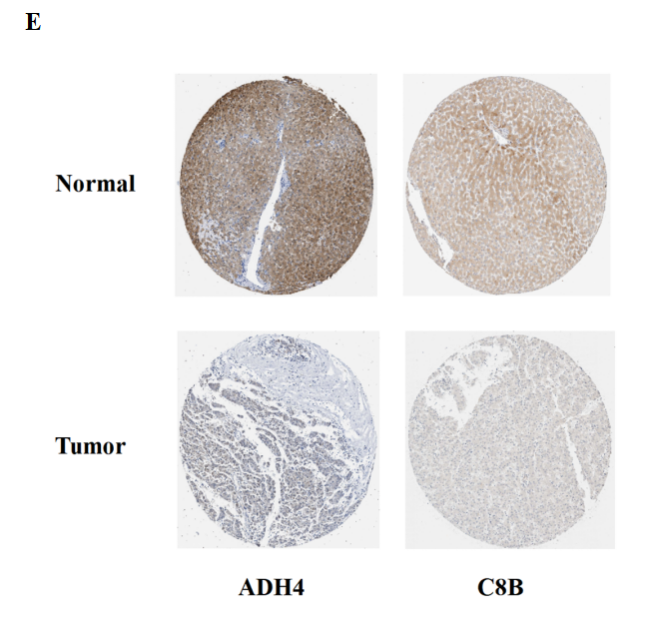
**

**Fig. S3** The results of immunohistochemistry of specific genes (ADH4 and C8B) were validated using the HPA database

**Fig. S4**


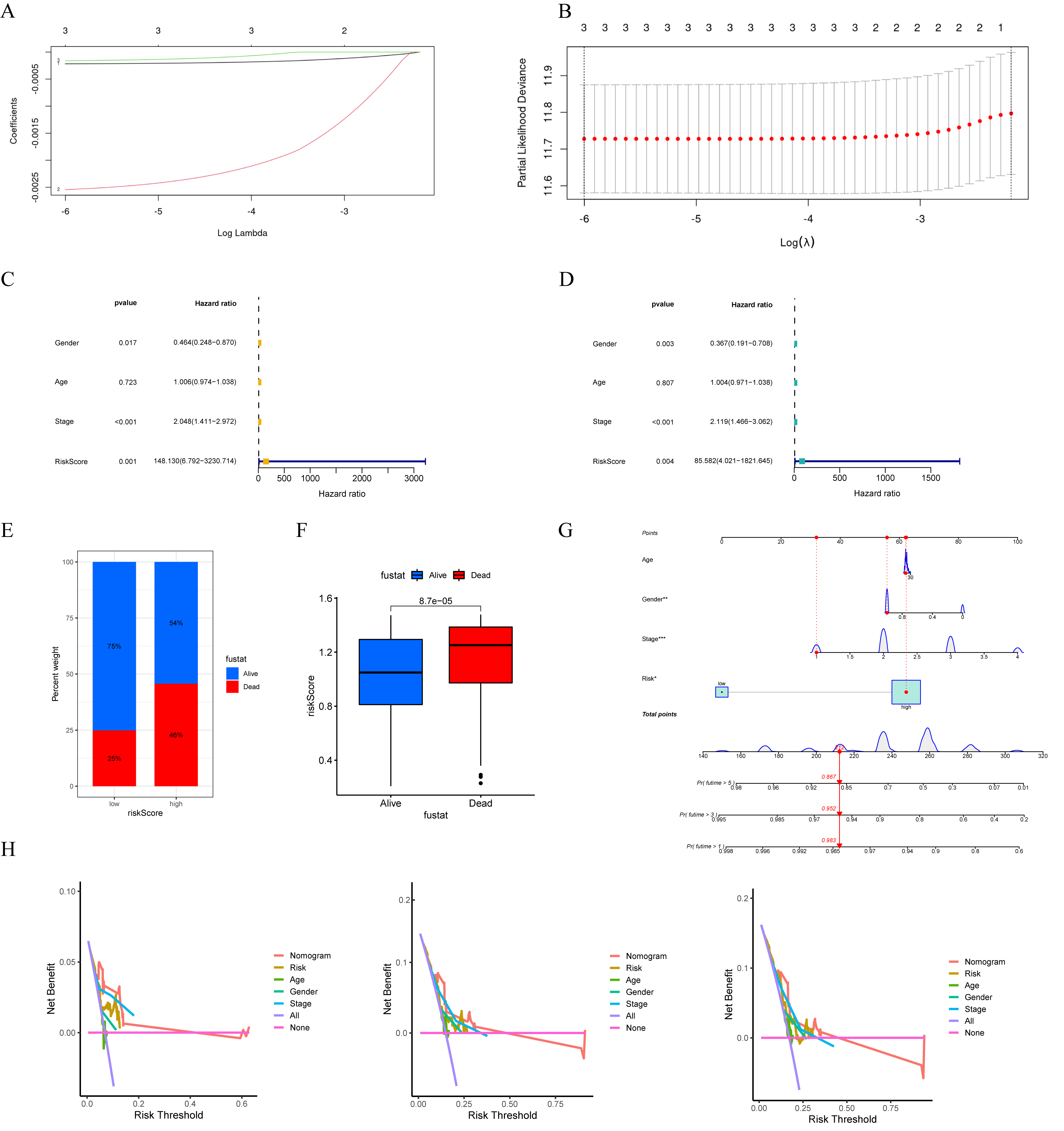


**Fig. S4** (**A**) Lasso regression analysis based on three specific genes. (**B**) The method of cross-validation was used to select the optimal genes. The forest plots were established based on the univariate (**C**) and multivariate (**D**) Cox regression analysis in the ICGC-LIRI-JP cohort. (**E**) Analysis of clinical survival status in different risk groups. (**F**) Correlation analysis of clinical survival status and the risk score. (**G**) The nomogram was created by integrating risk groups and clinical characteristics within the ICGC-LIRI-JP cohort. (**H**) Decision curve analysis of nomogram (1-, 2-, 3- year). * *P* < 0.05, ** *P* < 0.01, *** *P* < 0.001, *ns*: The difference is not significant

**Fig. S5**


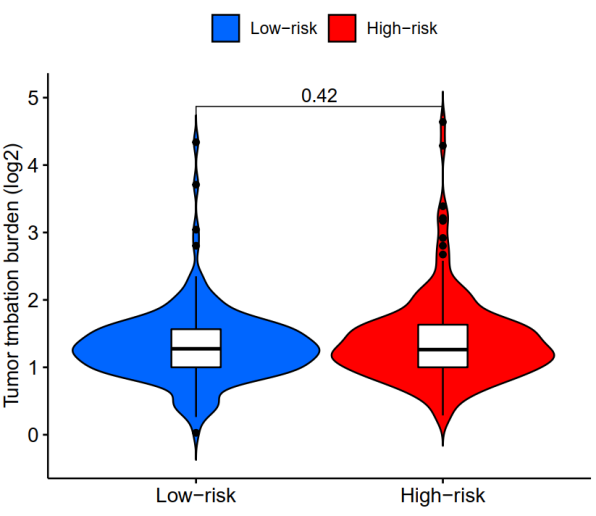


**Fig. S5** Variation analysis of the TMB in different risk groups, *P* < 0.05 was considered statistically significant
