# Supplementary material for: Identification and validation of a novel predictive signature based on hepatocyte-specific genes in hepatocellular carcinoma by integrated analysis of single-cell and bulk RNA sequencing
Source: BMC Med Genomics. 2024 Apr 23;17:103. doi: 10.1186/s12920-024-01871-1 (PMC11040759; doi:10.1186/s12920-024-01871-1)
Supplement: Supplementary file 2 — Supplementary Material 2 [file 12920_2024_1871_MOESM2_ESM.docx]

**Table S1** The genes list of the 98 intersecting genes

| **Gene Name** |
| --- |
| ADH4 |
| KIFC1 |
| SPP1 |
| CLIC1 |
| PTTG1 |
| ILF2 |
| LINC01554 |
| LCAT |
| H2AFZ |
| NOP58 |
| C19orf48 |
| CCT3 |
| ATP1B3 |
| C8B |
| KPNA2 |
| SNRPD1 |
| NEU1 |
| TMEM106C |
| HN1 |
| MPV17 |
| EIF4A3 |
| METTL5 |
| POLR2G |
| MARCKSL1 |
| HMGA1 |
| CCNB1 |
| UPB1 |
| AHSA1 |
| GYS2 |
| CBX1 |
| CES3 |
| TMEM147 |
| CDK1 |
| NAP1L4 |
| CCT7 |
| DTYMK |
| ITGB1BP1 |
| RCL1 |
| MCM7 |
| ATIC |
| LARS |
| TIMM23 |
| HILPDA |
| SF3B4 |
| HEXB |
| HDAC2 |
| VPS72 |
| UCK2 |
| SMS |
| CHORDC1 |
| APIP |
| MARC2 |
| MED10 |
| DUSP12 |
| ERCC1 |
| HM13 |
| YIF1B |
| GARS |
| NCBP2 |
| PPP2R1A |
| CFHR3 |
| COMMD3 |
| VPS26A |
| RTN3 |
| ETV5 |
| NUP37 |
| SF3A3 |
| PSMD13 |
| PHC2 |
| FAIM |
| RPUSD3 |
| GPATCH4 |
| DDX56 |
| GNL2 |
| MED8 |
| ISY1 |
| FANCL |
| ANXA10 |
| NUP85 |
| BRD8 |
| C20orf27 |
| USP39 |
| PAIP1 |
| BCL10 |
| RARS |
| MRTO4 |
| UPF3B |
| MITD1 |
| ELOVL1 |
| RNF8 |
| SLC9A3R1 |
| TMEM251 |
| CFHR4 |
| SNRPA |
| ERI3 |
| TARS |
| PLOD2 |
| EIF2B5 |
